# Investigation of rotameric conformations of substituted imidazo-[1,2-*a*]pyrazine: experimental and theoretical approaches†

**DOI:** 10.1039/c7ra13617j

**Published:** 2018-03-07

**Authors:** Gulshan Kumar, Richa Goel, Kamaldeep Paul, Vijay Luxami

**Affiliations:** School of Chemistry and Biochemistry, Thapar University Patiala-147004 India vluxami@thapar.edu

## Abstract

The different rotameric conformations of imidazo-[1,2-*a*]pyrazine have been synthesized and characterized by means of different experimental techniques, such as NMR, FTIR, and absorption spectroscopy and quantum chemical calculations. The different conformations were stabilized by hydrogen bonds, such as OH⋯N, ArH⋯N and ArH⋯ArH. The ground state optimizations and potential energy surface (PES) scanning profiles produced using density functional theory (DFT) show two stable rotameric forms for each molecule. The relative population of the conformations is affected by the strength of the hydrogen bonds. The calculated absorption spectra and isotopic shielding constants were acquired by time-dependent density functional theory (TD-DFT) and gauge invariant atomic orbitals (GIAO)-DFT, respectively. The strength of the hydrogen bonding interactions that resulted in the different conformations was studied by quantum theory of atoms in molecules (QTAIM).

## Abstract 2

The different rotameric conformations of imidazo-[1,2-*a*]pyrazine have been synthesized and characterized by means of different experimental techniques, such as NMR, FTIR, and absorption spectroscopy and quantum chemical calculations.

## Graphical abstract

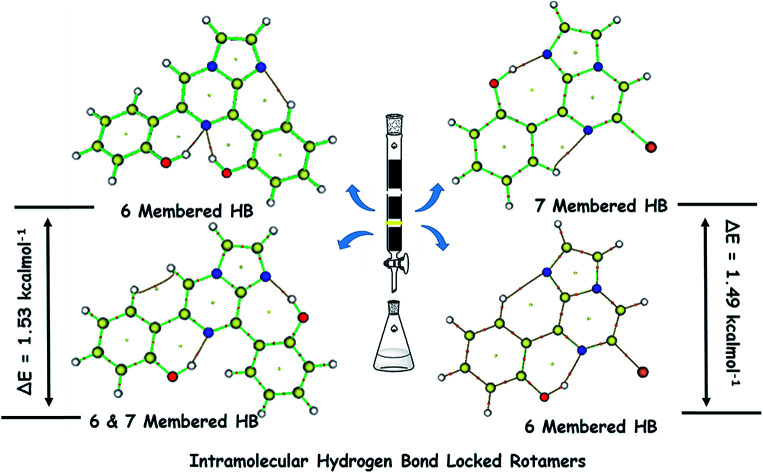

## 1. Introduction

The heterocyclic compounds are essential candidates in the chemical, biological, agricultural, and veterinary fields.^1,2^ Among the various heterocycles, imidazo[1,2-*a*]pyrazine is an aza-heterocyclic compound that has applications as an anticancer agent,^3^ cardiac stimulating agent and uterine relaxant,^4^ antihyperglycemic agent,^5^ antihypertensive agent,^6^ antiulcer agent,^7^ antibronchospastic agent^8^*etc.*,^9–11^ The properties of compounds as an application candidate depend upon the molecular structure of the compound and can be tuned by altering the molecular modification, and orientation of the compound.^12^

The spatial and chemical constraints in molecular systems give rise to different spatial arrangements or conformations. These conformations can be governed by non-covalent interactions, such as van der Waals interactions, hydrogen bonding, and steric hindrance significantly.^12–15^ These non-covalent interactions are the deciding forces in building supramolecular assemblies, and thus, this is a fascinating area in structural chemistry. These interactions connect together the synthons and building blocks of supramolecular structures.^12,15^ Among the noncovalent interactions, hydrogen bonds, such as C–H⋯O, N–H⋯O, O–H⋯N, C–H⋯π, O–H⋯π, and π–π interactions, have a conclusive role in controlling molecular conformations. Thus, the implication of such interactions in conformational analysis would result in new conformations of compounds in the solid, solution and gas phase. These interactions lock the molecular configurations through hydrogen bonding, and thus, permit the formation of different conformations.^16–24^ Thus, the exact structure determination of a molecule is important and a big subject of research itself in science. Structural determination requires tough conformational analysis by means of experimental and theoretical calculations.

For decades, correct structural information has been very helpful in describing many chemical, physical and biological processes, such as reaction mechanisms and photophysical processes, and in designing molecular machines and understanding their functioning, *etc.*^25,26^ The problem of exact structure determination is solved by experimental (*e.g.* spectroscopic techniques), single crystal X-ray diffraction (SCXRD) and theoretical (quantum chemical calculations) tools. Spectroscopic techniques, such as FTIR, NMR, absorption spectroscopy *etc.*, provide very specific information about functional groups and their orientation, and thus, they are promising tools in solving molecular structures. Also, SCXRD provides the exact molecular configuration following good structural refinement, but it requires high-quality crystals and growing good quality crystals is not always an easy task. Therefore, conformer assignment requires experimental data from different techniques, and data analysis exercises, and thus is always a challenging task through experimental techniques. Therefore, the use of computational tools is the best alternative to predict an accurate structure *via* different quantum chemical methods. Quantum chemical calculations can be used to predict the spectroscopic values for different possible structures. The correlation of experimental and theoretical results is also a good parameter to confirm the exact molecular structure. During a literature survey, we found some reports on the conformational examination of organic compounds by mean of experimental and theoretical techniques, but the conformation of imidazo-[1,2-*a*]pyrazine has not been determined.^27–32^ In this work, we have synthesized a series of imidazo-[1,2-*a*]pyrazine derivatives that exhibit different rotameric conformations. These rotameric conformations were further purified by column chromatography. Moreover, the rotamers were characterized through NMR, UV and quantum chemical calculations. Furthermore, the experimental data have been correlated with the calculated values to gain insight into the rotameric structures.

## 2. Experimental section

### 2.1. Material and methods

All the chemicals used for synthesis were purchased from Sigma-Aldrich Chemical Ltd., Loba Chemie *etc.* depending upon their availability. All the solvents used were of spectroscopic grade and purchased from Spectrochem and Rankem Ltd. All chemicals and solvents were used without further purification. The progress of the chemical reaction was monitored by thin-layer chromatography (TLC). Melting points were recorded using the open capillary tube method and were uncorrected. ^1^H NMR and ^13^C NMR spectra were recorded on a JEOL ECS-400 MHz spectrometer at ambient temperature in CDCl_3_ with TMS as an internal reference. All chemical shifts were reported in ppm relative to the reference. Mass spectra of the synthesised compounds were recorded using a Waters Micromass Q-Tof micro mass spectrometer. The absorption spectra were recorded on a SHIMADZU-2600 spectrophotometer using quartz cuvettes of 1 cm path length.

### 2.2. Synthesis

**Scheme 1:**
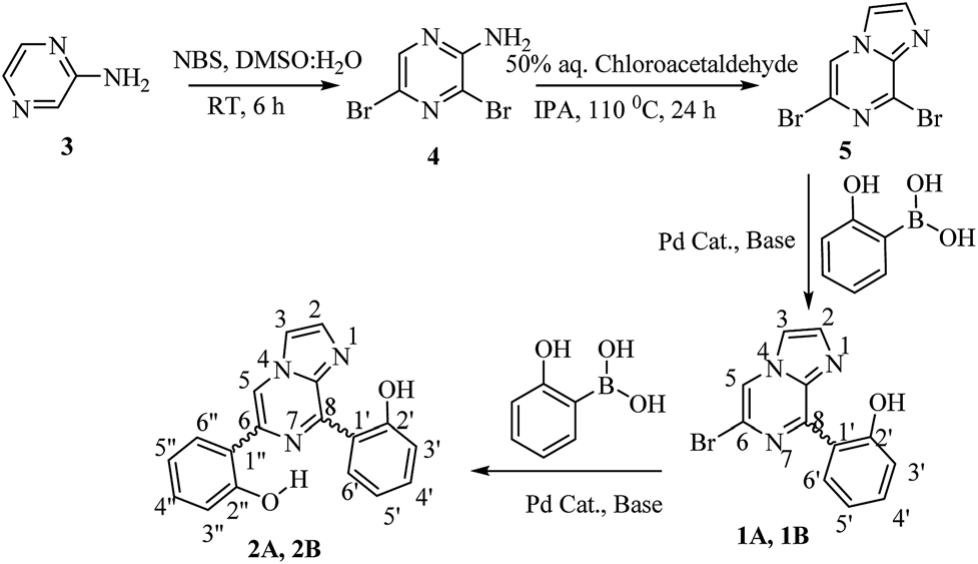
Synthesis of compounds 1 and 2.

2-Aminopyrazine was used as the starting material, which was brominated with *N*-bromosuccinimide (NBS) in DMSO and H_2_O at room temperature for 6 hours. The resultant 2-amino-3,5-dibromopyrazine (4)^33^ was cyclized with chloroacetaldehyde in iso-propyl alcohol to obtain 6,8-dibromo-imidazo[1,2-*a*]pyrazine (5). Subsequently, a Suzuki–Miyaura cross-coupling reaction was performed with 6,8-dibromo-imidazo[1,2-*a*]pyrazine and 2-hydroxyphenyl boronic acid in the presence of a palladium catalyst and afforded the substituted mono- and di-arylated products (1 and 2, Scheme 1).^34^

### 2.3. Computational details

All theoretical calculations were performed using the GAUSSIAN-03W program.^35^ The geometry optimizations for all molecules were carried out at the DFT/B3LYP level of theory using the 6-31++G(d,p) basis set.^36–38^ Frequency calculations were performed on the obtained optimized structures at the same level of theory. The potential energy surfaces for the dihedral angles were acquired using DFT at the B3LYP/6-31+g* level of theory to identify the local minima. The theoretical absorption spectra were calculated from the vertical excitation energies using the time-dependent density functional theory (TD-DFT) method. The NMR shielding tensors were calculated using the GAIO method.^39^ The conformational optimization was performed in different solvents (CH_3_CN and CHCl_3_) for the optimized structures using a self-consistent reaction field (SCRF) approach coupled with the integral equation formation of polarizable continuum model (IEFPCM) solvation method. Topological analysis of the electron distribution was conducted on selected conformations according to Bader's “Quantum Theory of Atoms in Molecule (QTAIM)”.^40^ QTAIM calculations were carried out using the Multiwfn program^41^ to evaluate the nature and strength of the hydrogen bonds present in the different conformations.

## 3. Results and discussion

### 3.1. Synthesis of 2-amino-3,5-dibromopyrazine (4)^33^

*N*-Bromosuccinamide (14.95 g, 83.99 mmol) was added over 50 min to a mixture of 2-aminopyrazine (3.80 g, 40 mmol) in 80 ml of DMSO and 2 ml of H_2_O below 15 °C. The mixture was then stirred for 6 h at room temperature. After completion of the reaction, the mixture was extracted with water and ethyl acetate. The ethyl acetate layer was dried over Na_2_SO_4_ and concentrated under vacuum. The crude product was purified by column chromatography using hexane : ethyl acetate (9 : 1) as an eluent. White solid; yield: 90%; mp 115–116 °C; ^1^H NMR (CDCl_3_, 400 MHz): *δ* 5.12 (bs, 1H, NH_2_), 8.04 (s, 1H, C6H); ^13^C NMR (CDCl_3_, 100 MHz): *δ* 123.5, 123.9, 143.1, 151.8; MS (EI): *m*/*z* 254 (M^+^ + 1).

### 3.2. Synthesis of 6,8-dibromo-imidazo[1,2-*a*]pyrazine (5)^34^

To 2-amino-3,5-dibromopyrazine (5.0 g, 19.8 mmol) in 100 ml of isopropyl alcohol (IPA), a 50% aqueous solution of chloroacetaldehyde (99 mmol) was added dropwise. The reaction mixture was refluxed at 110 °C for 24 h. After the completion of the reaction, the reaction mixture was cooled to room temperature and then extracted with water and chloroform. The chloroform layer was dried over sodium sulphate and concentrated under vacuum to get the crude product. The product was purified by column chromatography using hexane : ethyl acetate (6 : 4) as an eluent to obtain a pure compound as a white solid. Yield: 80%; mp: 163–165 °C; ^1^H NMR (CDCl_3_, 400 MHz): *δ* 7.80 (d, *J* = 0.92 Hz, 1H, C2H), 7.86 (d, *J* = 1.36 Hz, 1H, C3H), 8.29 (s, 1H, C5H); ^13^C NMR (CDCl_3_, 100 MHz): *δ* 115.9, 119.3, 119.9, 137.0, 137.4, 142.5; MS (EI): *m*/*z* 278 (M^+^ + 1).

### 3.3. Synthesis of compound 1

A vial equipped with a stirring bar was charged with 6,8-dibromo-imidazo[1,2-*a*]pyrazine (5) (0.5 g, 1.8 mmol), Cs_2_CO_3_ (0.6 g, 1.8 mmol) and boronic acid (1.8 mmol), dissolved in CH_3_CN : H_2_O (9 : 1) at 100 °C under an inert atmosphere. Then, 5 mol% of Pd(PPh_3_)_4_ was added and the vial was capped. The reaction mixture was refluxed for 7–12 h. After the completion of the reaction (monitored by TLC), the reaction mixture was cooled, and then extracted with water and chloroform. The organic layer was dried over Na_2_SO_4_, filtered and concentrated under vacuum to obtain the crude product. The residue was purified by silica gel (60–120 mesh) column chromatography using hexane : ethyl acetate as an eluent. Two different coloured solid products, 1A and 1B, were obtained.

#### 1A

Yellow solid; yield: 30%; mp 232–234 °C; ^1^H NMR (CDCl_3_, 400 MHz): *δ* 7.05–7.09 (m, 2H, ArH), 7.42–7.47 (m, 1H, ArH), 7.74 (s, 1H, C2H), 7.90 (s, 1H, C3H), 8.23 (s, 1H, C5H), 9.54–9.56 (m, 1H, ArH), 13.03 (s, OH); ^13^C NMR (CDCl_3_, 100 MHz): *δ* 114.7, 117.2, 117.9, 118.5, 118.9, 119.4, 132.4, 133.7, 135.8, 137.4, 150.0, 160.8 (ArC); MS (EI): *m*/*z* 292 (M^+^ + 1).

#### 1B

White solid; yield: 30%; mp 232–234 °C; ^1^H NMR (CDCl_3_, 400 MHz): *δ* 7.29–7.34 (m, 2H, ArH), 7.43–7.47 (m, 2H, ArH), 7.68 (d, *J* = 1.40 Hz, 1H, C2H), 7.77 (d, *J* = 0.92 Hz, 1H, C3H), 7.99 (s, 1H, C5H); ^13^C NMR (CDCl_3_, 100 MHz): *δ* 115.3, 115.5, 119.3, 121.8, 125.9, 129.6, 132.6, 135.2, 152.1, 152.1 (ArC); MS (EI): *m*/*z* 291 (M^+^ + 1).

### 3.4. Synthesis of compound 2

A vial equipped with a stirring bar was charged with 6-bromo-8-substituted-imidazo[1,2-*a*]pyrazine (1A or 1B) (0.1 g, 0.323 mmol), Cs_2_CO_3_ (0.105 g, 0.323 mmol) and 2-hydroxyphenylboronic acid (0.323 mmol), dissolved in CH_3_CN : H_2_O (9 : 1) at 100 °C under an inert atmosphere. Then, 5 mol% of Pd(PPh_3_)_4_ was added, the vial was capped and the mixture was refluxed for 6–12 h. After the completion of the reaction (monitored by TLC), the reaction mixture was cooled, and then extracted with water and chloroform. The organic layer was dried over Na_2_SO_4_, filtered and concentrated under vacuum to obtain the crude product. The residue was purified by silica gel (100–200 mesh) column chromatography using hexane : ethyl acetate as an eluent. Two new different compounds, namely 2A and 2B, were obtained with respect to their starting reactants 1A and 1B.

#### 2A

Yellow solid; yield: 65%; mp 240–241 °C; ^1^H NMR (CDCl_3_, 400 MHz): *δ* 6.97 (t, *J* = 7.48 Hz, 1H, ArH), 7.05 (d, *J* = 8.52 Hz, 1H, ArH), 7.10 (t, *J* = 7.48 Hz, 1H, ArH), 7.17 (d, *J* = 7.48 Hz, 1H, ArH), 7.31–7.35 (m, 1H, ArH), 7.46–7.50 (m, 1H, ArH), 7.65 (d, *J* = 6.52 Hz, 1H, ArH), 7.87 (s, 1H, C2H), 7.88 (s, 1H, C3H), 8.27–8.30 (m, 1H, ArH), 8.59 (s, 1H, C5H); ^13^C NMR (CDCl_3_, 100 MHz): *δ* 113.3, 115.2, 117.8, 118.6, 119.9, 120.8, 121.4, 125.7, 130.9, 131.2, 133.6, 133.7, 137.6, 139.8, 147.9, 157.6, 158.0 (ArC); MS (EI): *m*/*z* 303 (M^+^ + 1).

#### 2B

White solid; yield: 70%; mp 244–246 °C; ^1^H NMR (CDCl_3_, 400 MHz): *δ* 6.82–6.86 (m, 2H, ArH), 7.18 (t, *J* = 7.56 Hz, 1H, ArH), 7.29–7.31 (m, 2H, ArH), 7.39 (t, *J* = 7.34 Hz, 1H, ArH), 7.50–7.55 (m, 2H, ArH), 7.80 (s, 1H, C2H), 7.84 (s, 1H, C3H), 8.28 (s, 1H, C5H), 10.70 (s, 1H, OH); ^13^C NMR (CDCl_3_, 100 MHz): *δ* 110.5, 116.2, 117.1, 118.9, 119.6, 122.1, 124.9, 126.8, 130.3, 130.9, 131.7, 135.6, 136.5, 151.9, 152.6, 157.4 (ArC); MS (EI): *m*/*z* 304 (M^+^ + 1).

Interestingly, the molecular mass from ESI-MS was found to be the same for compounds 1A and 1B (Fig. S3, and S7†), and likewise for 2A and 2B (Fig. S14, and S19†), but with different NMR signals (Fig. S1, S8, S5, S6, S12, S13, S17 and S18†). Furthermore, all the synthesized compounds have different absorption maxima (Fig. S10 and S21†), and characteristic FTIR signals (Fig. S4, S8, S15 and S20†), which clearly indicates that the obtained compounds 1A and 1B are isomeric forms of each other, as are 2A and 2B. The obtained compounds have a rigid architecture, except for rotation around the single bond connecting the phenyl ring to the imidazo-[1,2-*a*]pyrazine moiety. Therefore, the only possibility is for the isomeric forms to be different rotameric conformations. Thus, in order to determine the accurate structures of the possible conformers of compounds 1 and 2, theoretical calculations were performed.

### 3.5. Geometry optimization and reactivity of compound 1

**Fig. 1:**
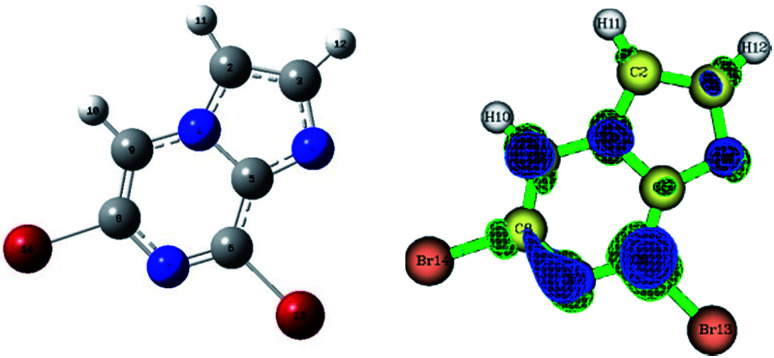
Optimized structure of the precursor molecule (5) (left) and the Fukui function map (right) (isosurface = 0.05), where the green and blue regions denote positive and negative electron density, respectively.

Compound 1 was optimized, and was further subjected to Fukui indices analysis, which is widely used to predict the reactivity indices in order to observe the reactive sites (electrophilic and nucleophilic sites) present on a molecule.^42^ Fukui functions are the partial derivatives of electron density with respect to the number of electrons present.
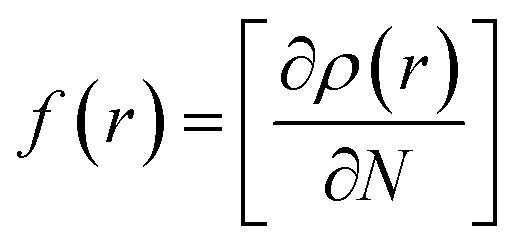
where *ρ*(*r*) denotes the electron density, and *N* is the number of electrons present in the molecular system. Generally, the indices are calculated for the HOMO and LUMO, and represent the nucleophilicity and electrophilicity index, respectively. Reactive sites in the molecular system have larger Fukui index values (Fig. 1).

#### 3.5.1. Conformational analysis of compound 1

The aim of finding the most stable conformation has been fulfilled using a simple general strategy.

**Scheme 2:**
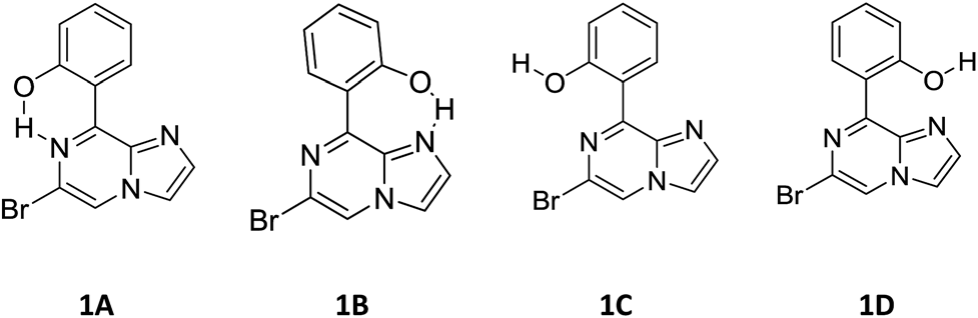
Possible conformations for compound 1.

1. Compound 1 has rotational flexibility with respect to the single bond joining the hydroxy phenyl ring and the rigid imidazo-[1,2-*a*]pyrazine ring. Therefore, a conformational search was carried out using the rotational potential energy surface (PES) produced by DFT at the B3LYP/6-31+G* level. All possible conformations of compound 1 at local minima are shown in Scheme 2.

2. The obtained structures at local minima were analyzed to determine their relative Boltzmann contributions. From the Boltzmann distribution principle, any conformers with a free energy in the region of 5 kcal mol^−1^ can make some contribution to the Boltzmann population at room temperature (RT). Thus, the contributing conformers have been further optimized using DFT at the B3LYP/6-31++G(d,p) level and confirmed as having no imaginary frequencies.

3. The absorption spectra and NMR chemical shifts were computed for each of the optimized conformers using the TD-DFT and GIAO methods.

4. The experimental and calculated data for the absorption spectra, FTIR signals, and NMR shielding tensors were compared and evaluated to determine the structure of the compounds.

#### 3.5.2. Structural analysis of compound 1

**Fig. 2:**
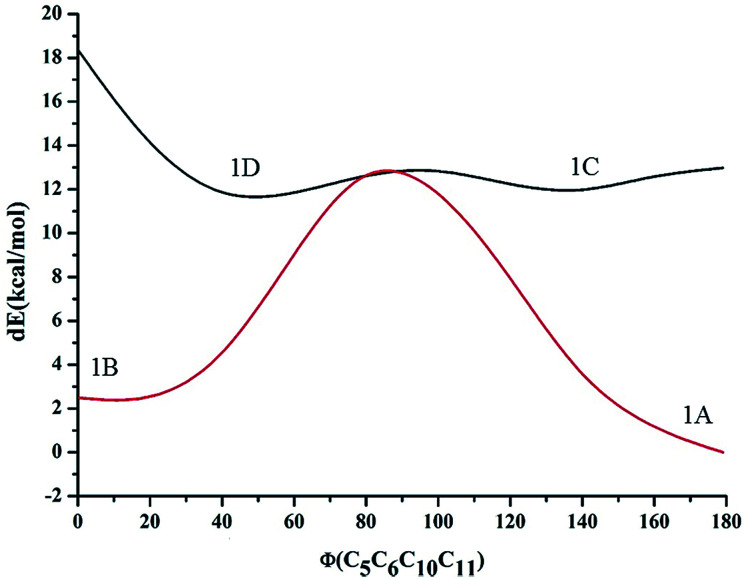
Energy variation (in kcal mol^−1^) of the different conformations for compound 1 along with the dihedral angle between the imidazole moiety and the phenolic moiety in the ground state.

**Fig. 3:**
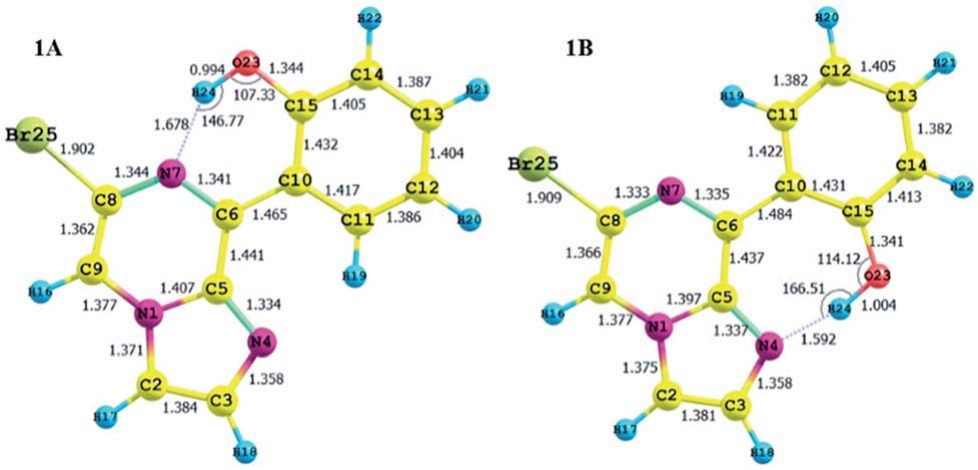
Optimized structures of the stable conformers for compound 1, along with their structural parameters.

At first, compound 1 was optimized in the form represented by 1A. Then, the potential energy surface (PES) of the dihedral angle for rotation of the single bond connecting the phenyl ring and imidazo-[1,2-*a*]pyrazine moiety was acquired for 1A. The PES resulted in a new local minimum, with the conformation represented by 1B. Both conformers were found to be stabilized by hydrogen bonding, primarily examined by looking at the bond distance of OH and NH, and it was found that the conformer at the local minimum 1B has an energy close to that of 1A. On the other hand, compound 1 was also optimized as conformer 1C to determine the impact of hydrogen bonding in the stabilization of conformers 1A and 1B. Also, the dihedral angle PES was analyzed to check for other local minima. The new local minimum observed was assigned as conformer 1D. The relative energy diagram for all conformations is shown in Fig. 2. It is found that both 1C and 1D have high energy relative to 1A and 1B. The relative free energies of the optimized conformations, along with their Boltzmann distributions, are summarized in Table 1. From the Boltzmann distribution principle, conformers with a free energy in the region of 5 kcal mol^−1^ can make some contribution to the Boltzmann population at room temperature (RT). Furthermore, conformers 1A and 1B have been optimized at the B3LYP/6-31++G(d,p) level. Both conformers, 1A and 1B, were found to exhibit a planar geometry and be stabilized by six-membered and seven-membered hydrogen bonding, respectively. Conformer 1A is the major product with an abundance of about ∼92.5%, while 1B, which is close in energy to conformation 1A, makes a relatively low (∼7.5%) contribution. No other conformations make a significant contribution. The optimized structures of the conformations are shown in Fig. 3, along with important structural parameters, such as bond lengths and bond angles. The stabilization of the conformations is discussed below:

**Table 1:** The relative free energies of the optimized conformations along with the Boltzmann distributions for compound 1

Conformer	Δ*E* (kcal mol^−1^)	Boltzmann distribution (%)
1A	0.000	92.5
1B	1.488	7.5
1C	9.948	0
1D	23.845	0

#### 3.5.3. Conformer 1A

According to the Boltzmann distribution and optimized geometry, conformer 1A is the key conformation in the gas phase. The stability of this conformation can be explained by the relative charges and hydrogen bond distances.

The calculated charges on the H24 [0.505 (NBO)], N7 [−0.571 (NBO)] and O23 [−0.677 (NBO)] atoms establish an intramolecular dipolar interaction between a phenolic hydrogen (O23–H24) and the pyrazine nitrogen (N7) of the imidazole moiety.

The calculated non-bonded distance between N7 and H24 bonded to O23 [*d* (N7⋯H24) = 1.678 Å] is less than the sum of the van der Waals radii of hydrogen and nitrogen. The smaller distance and columbic interaction with opposite charges on H24 and N7 support six-membered intramolecular hydrogen bonding.

#### 3.5.4. Conformer 1B

The other probable conformer, 1B, has a relatively low Boltzmann contribution in spite of its planar geometry.

The calculated charges on the H24 [+0.520 (NBO)], N4 [−0.539 (NBO)] and O23 [−0.560 (−0.712 (NBO))] atoms establish an intramolecular dipolar interaction between a phenolic hydrogen (O23–H24) and the pyrrole nitrogen (N4).

The non-bonded distance between the phenolic hydrogen and pyrrole nitrogen [*d* (H24⋯N4) = 1.592 Å], which is less than the sum of the van der Waals radii of hydrogen and nitrogen, supports hydrogen bonding. The smaller distance and columbic interaction with opposite charges on H24 and N4 confirmed the seven-membered intramolecular hydrogen bonding.

In addition to the N⋯H–O hydrogen bonding interactions in conformers 1A and 1B, there are ArH⋯N interactions, where the aromatic hydrogen of the phenolic moiety interacts with the pyrrole nitrogen for conformer 1A and the imidazole nitrogen for conformer 1B. These interactions are weak in nature, as confirmed by the interaction distances and topological parameters. Thus, conformer 1B exhibits seven-membered intramolecular hydrogen bonding, which is less stable than the six-membered hydrogen bonding exhibited by conformer 1A, and this could be the reason for the low Boltzmann contribution for conformer 1B.

Additionally, both conformers 1A and 1B were free from interelectronic repulsion, which is present in conformers 1C and 1D. Furthermore, the hydrogen bonding interaction was also estimated by QTAIM calculations using the Multiwfn program (Table 2).

**Table 2:** Topology parameters, including electron density (*ρ*), Laplacian electron density (∇^2^*ρ*), potential energy density [*V*(*r*)], total energy density [*H*(*r*)], and hydrogen bonding energy [*E*_HB_(kcal mol^−1^)] at the bond critical point of non-covalent interactions (D⋯HA) for conformers 1A and 1B at the B3LYP/6-31++G** level

Compound	Interactions	BCP	*d* (Å)	∠D⋯HA	∇^2^*ρ*	*V*(*r*)	*G*(*r*)	*H*(*r*)	*E*_HB_
1A									
	N⋯HO	(3, −1)	1.68	146.77	0.1303	−0.0452	0.0419	−0.0063	−14.18
	ArH⋯N	(3, −1)	2.19	129.10	0.0637	−0.0131	0.0145	−0.0014	−4.11

1B									
	N⋯HO	(3, −1)	1.59	166.51	0.1295	−0.0581	0.0453	−0.0129	−18.22
	ArH⋯N	(3, −1)	2.25	102.36	0.0812	−0.0137	0.0170	−0.0033	−4.39

#### 3.5.5. Topology parameters

**Fig. 4:**
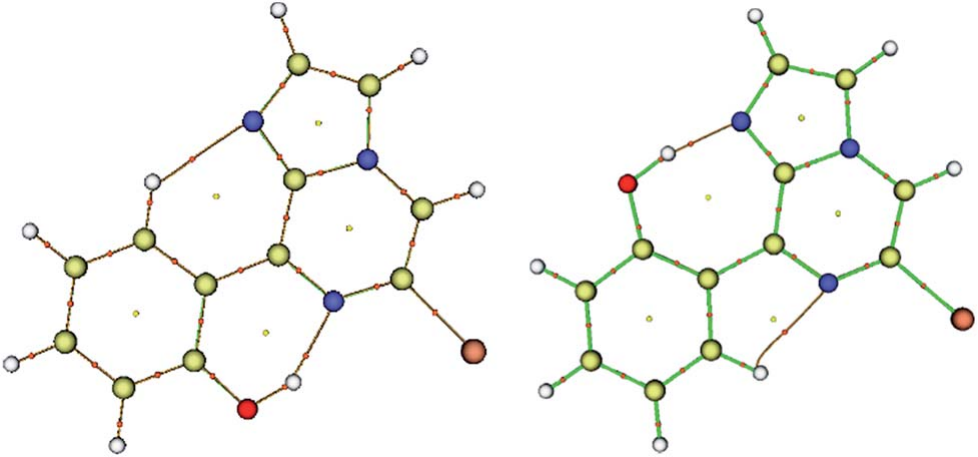
Molecular graphs for conformers 1A (left) and 1B (right); the red dots denote the bond critical points along the bond paths.

In order to determine the nature of the intramolecular hydrogen bonds (HBs) existing within the key conformers, they were studied by means of Bader's quantum theory of atoms in molecules (QTAIM) using Multiwfn 3.3.7. The existence of hydrogen bonding was examined based on Popelier criteria.^43^ These criteria are (1) the occurrence of a critical point (CP) between two neighboring atoms, (2) the electron density [*ρ*(*r*_c_)] being in the range of 0.002–0.034 au and (3) the Laplacian of the electron density [∇^2^*ρ*(*r*_c_)] being in the range of 0.024–0.139 au at the critical point. The topological properties of the bond critical points (BCPs) were examined for the stability and strength of the interaction (Table 2). The positive value of the Laplacian of the electron density [∇^2^*ρ*(*r*_c_)] for both conformers 1A and 1B, indicated electrostatic closed shell interactions and the value was found to be within Popelier's hydrogen bonding criteria. The Laplacian of the electron density for N⋯HO hydrogen bonding in conformer 1A is higher than that in 1B (Table 2). On the other hand, ArH⋯N has more Laplacian electron density in conformer 1B. Furthermore, the degree of covalency and strength of the interactions were characterized according to Rozas' rules: (i) ∇^2^*ρ*(*r*_c_) < 0 and *H*(*r*) < 0 for strong H-bonds of covalent nature, (ii) ∇^2^*ρ*(*r*_c_) > 0 and *H*(*r*) < 0 for medium H-bonds of partially covalent nature, and (iii) ∇^2^*ρ*(*r*_c_) > 0 and *H*(*r*) > 0 for weak H-bonds of electrostatic character.^30^ All of the bond critical points in Table 2 (∇^2^*ρ*(*r*_c_) > 0 and *H*(*r*) < 0) are designated as medium range interactions with partial covalent character for both conformers. Moreover, the hydrogen bond energy is determined using the Espinosa equation,^44^ which states that the interaction energy of a H⋯X contact is defined as *E*_HX_ = *V*(*r*)/2 at the BCP, where *V*(*r*) is the potential electron density at the bond critical point. Conformer 1A exhibited two interactions, N⋯HO and ArH⋯N, which contributed −14.18 and −4.11 kcal mol^−1^ of stabilization. On the other hand, conformer 1B also exhibited N⋯HO and ArH⋯N interactions, which contributed −18.22 and −4.39 kcal mol^−1^ of stabilization, respectively. The ArH⋯N interactions make nearly the same contribution for both conformers 1A and 1B, while the N⋯HO hydrogen bonds were found to be weaker in conformer 1A than 1B. This is due to the presence of an electron withdrawing bromine atom, which attracts the electron density, and therefore weakens the hydrogen bonding. On the other hand, the lone pair on the nitrogen atom could easily interact with a polarized hydrogen atom in conformer 1B, forming strong hydrogen bonding. The bond critical points and bond paths of conformers 1A and 1B are shown in Fig. 4.

#### 3.5.6. MBO and LBO analysis for compound 1

In addition, the Laplacian bond order (LBO) and Mayer bond order (MBO) were analyzed for stable conformers 1A and 1B. LBO has a direct correlation with the bond polarity and vibrational frequency. Bonds with a bond order value smaller than 1.0 are polarized in nature, and the lower the value, the greater the polarization. The Mayer bond order and Laplacian bond order are summarized in Table 3. The MBO for C15–O23 in conformer 1A is slightly lower than that for conformer 1B. On the other hand, a smaller value of MBO and LBO for O23–H24 reflects more bond polarity for conformer 1B than conformer 1A. The polarization of the OH bond causes a difference in bond strength, which results in a shift in the vibrational frequency of the two different conformations and validates the intramolecular hydrogen bonding.

**Table 3:** Effective bond order for conformers 1A and 1B

Bond	MBO at 6-31G**		LBO at 6-31++G**	
	1A	1B	1A	1B
N4–H24	—	0.1867	—	0.0011
N7–H24	0.1497	—	0.0006	—
C15–O23	1.0574	1.0746	0.5981	0.5917
O23–H24	0.7432	0.7011	0.5103	0.4638

#### 3.5.7. Frequency analysis

FTIR is a good tool for functional group analysis. The FTIR signals of functional groups including OH, C=N, C=C *etc.* were observed for both conformers 1A and 1B. All computed band positions are in good agreement with the experimental values for both conformers. However, the OH stretching vibrations, which are usually environmentally dependent, suggestively distinguish both conformations. The free O–H stretching vibration occurs in the range of 3500–3700 cm^−1^, and the hydrogen bonded O–H stretching vibration is usually observed in the range of 3200–3600 cm^−1^.^45,46^ The computed frequency results showed the O–H stretching band at 3230.25 cm^−1^ in FTIR for conformer 1A. Experimentally a broad O–H stretching band is observed at 3158 cm^−1^ for conformer 1A (Fig. S4†). The computed and experimental FTIR values for conformer 1B were at 2999.47 cm^−1^ and 3069 cm^−1^, respectively (Fig. S8†). The broadness in the band also confirmed the presence of intramolecular hydrogen bonding. The difference in the FTIR values of the O–H bands for 1A and 1B revealed that the OH bond was slightly weak in conformer 1A relative to 1B. As discussed earlier, the more polarized nature of the O–H bond as indicated by LBO bond order analysis and the strong hydrogen bonding indicated by QTAIM analysis for conformer 1B further weaken the O–H bond strength for 1B. Therefore, the FTIR values were in good agreement with the observations from topology analysis, hydrogen bond strength, and LBOs.

#### 3.5.8. NMR analysis

**Fig. 5:**
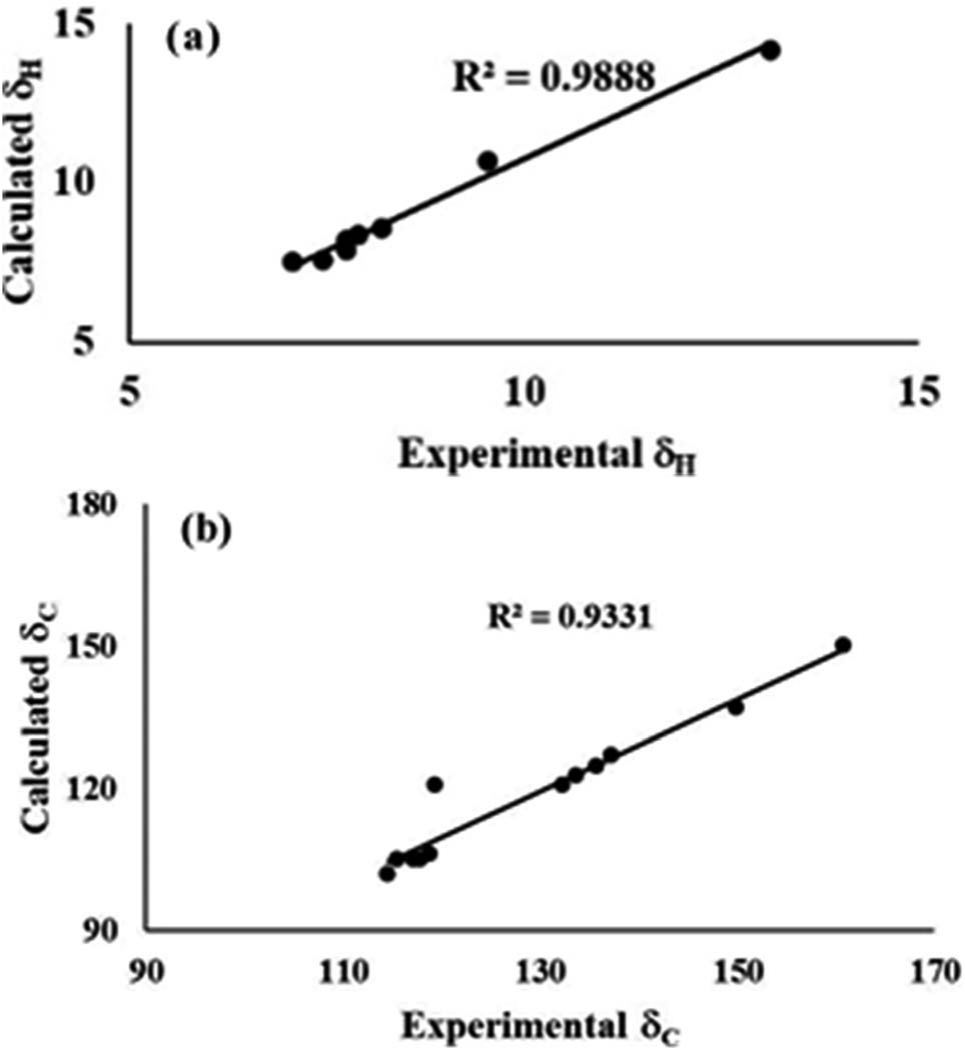
Experimental and theoretical correlation of (a) ^1^H and (b) ^13^C NMR for conformer 1A.

The ^1^H and ^13^C NMR shielding tensors were calculated using the GIAO method for the optimized structures, and the effect of the solvent was included using the IEFPCM model provided in Gaussian 03 W. The experimental chemical shifts of ^1^H NMR and ^13^C NMR were located between 7.07 and 13.12 ppm, and 114.65 and 160.84 ppm, respectively. The comparison between the computed and experimental ^1^H NMR and ^13^C NMR results gave correlation values of *R*^2^ = 0.9888 and *R*^2^ = 0.9331, respectively, for conformer 1A (Fig. 5). The deviation in the experimental chemical shift of C11 from the computed one is only due to the attachment of the hydroxyl group to the phenyl ring. In a similar way, the experimental NMR of conformer 1B has been compared with the calculated data. Although the correlation was not good, the combined information from FTIR and NMR could distinguish the two conformers. The correlation of the experimental and calculated NMR spectra for conformer 1B is shown in Fig. S9.† Also, it has been observed that the hydrogen atoms involved in hydrogen bonding resonate at different strengths for both 1A and 1B. The aromatic proton at C6′ involved in hydrogen bonding resonates at 9.54 ppm for compound 1A, while it resonates at 7.99 in the case of compound 1B (Table S1†).

#### 3.5.9. Absorption analysis

**Fig. 6:**
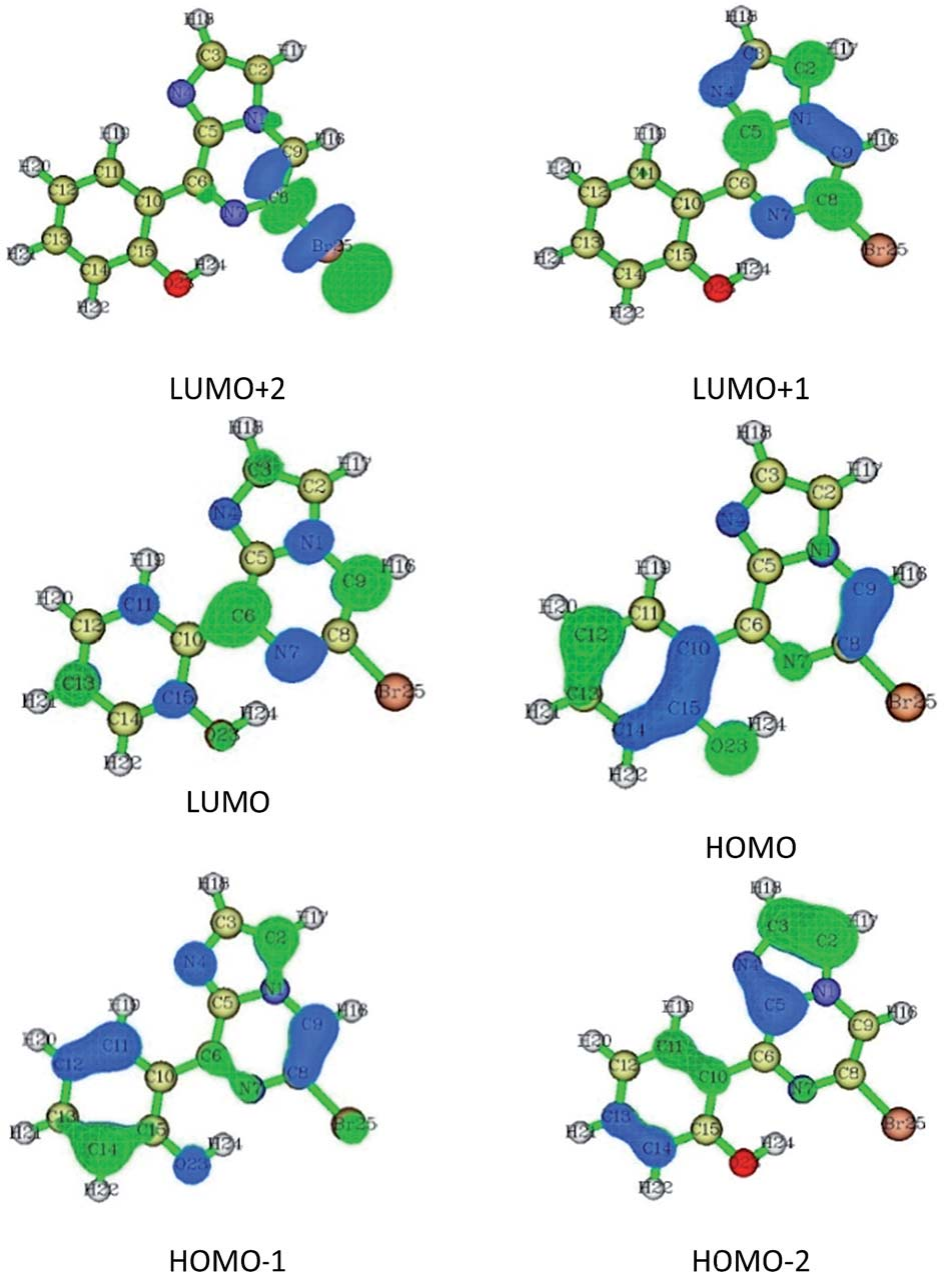
Frontier molecular orbitals for conformer 1A.

The vertical excitations were calculated for the optimized structures of conformers 1A and 1B in the gaseous and acetonitrile solution phases using TD-DFT. The obtained absorption value for conformation 1A at 380, 361, and 318 nm has a contribution from the first three Franck–Condon excited states [(S_0_–S_1_), (S_0_–S_2_), and (S_0_–S_3_)], respectively. The first transition results primarily from the HOMO to the LUMO, and has the nature of charge transfer from the HOMO to LUMO (from the phenolic moiety to the imidazole moiety), while the second and third transitions are kind of π–π* in nature and have major contributions from (HOMO-1) to LUMO and (HOMO-2) to LUMO transitions, respectively. On the other hand, the experimental absorption maximum value for conformer 1B was observed to be 290 nm, found due to the third Franck–Condon transition. The transition occurred from (HOMO-2) to LUMO due to charge transition in the opposite direction. The calculated and experimental absorption values for conformers 1A and 1B are summarized in Table S2† and the contributing Frontier molecular orbitals are shown in Fig. 6 and S11,† respectively.

Thus, the synthesized conformers have been optimized in the form of 1A and 1B. Both conformers 1A and 1B have a planar geometry and were found to be stabilised by six- and seven-membered hydrogen bonding, respectively. The stability and strength of the hydrogen bonds were established by QTAIM. The observed and calculated spectral data for NMR and FTIR were found to be in good correlation for each conformer.

### 3.6. Structural analysis of compound 2

**Scheme 3:**
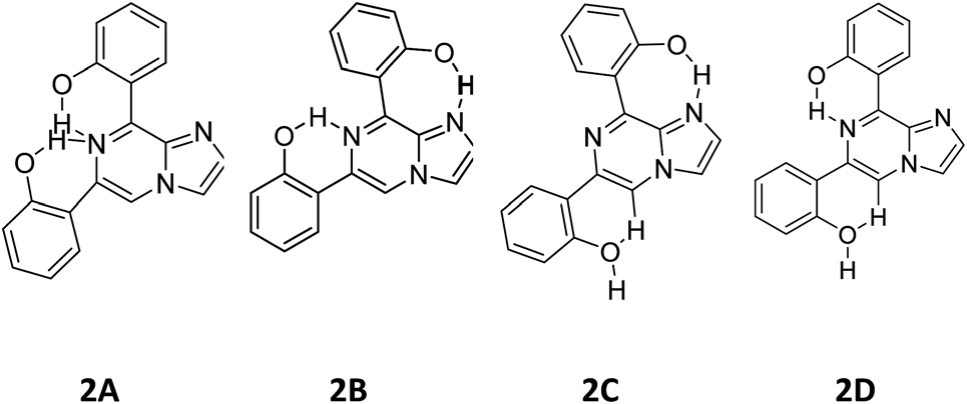
Possible conformations for compound 2.

**Fig. 7:**
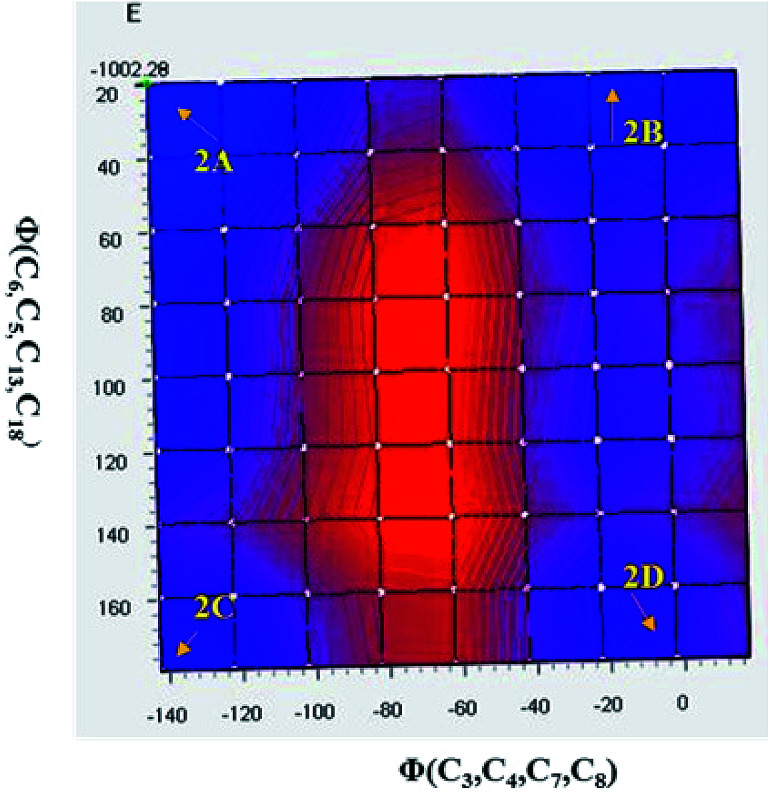
Potential energy surface for the dual dihedral angle between the imidazole moiety and a phenolic moiety in the ground state for compound 2, with four conformers at local minima.

**Fig. 8:**
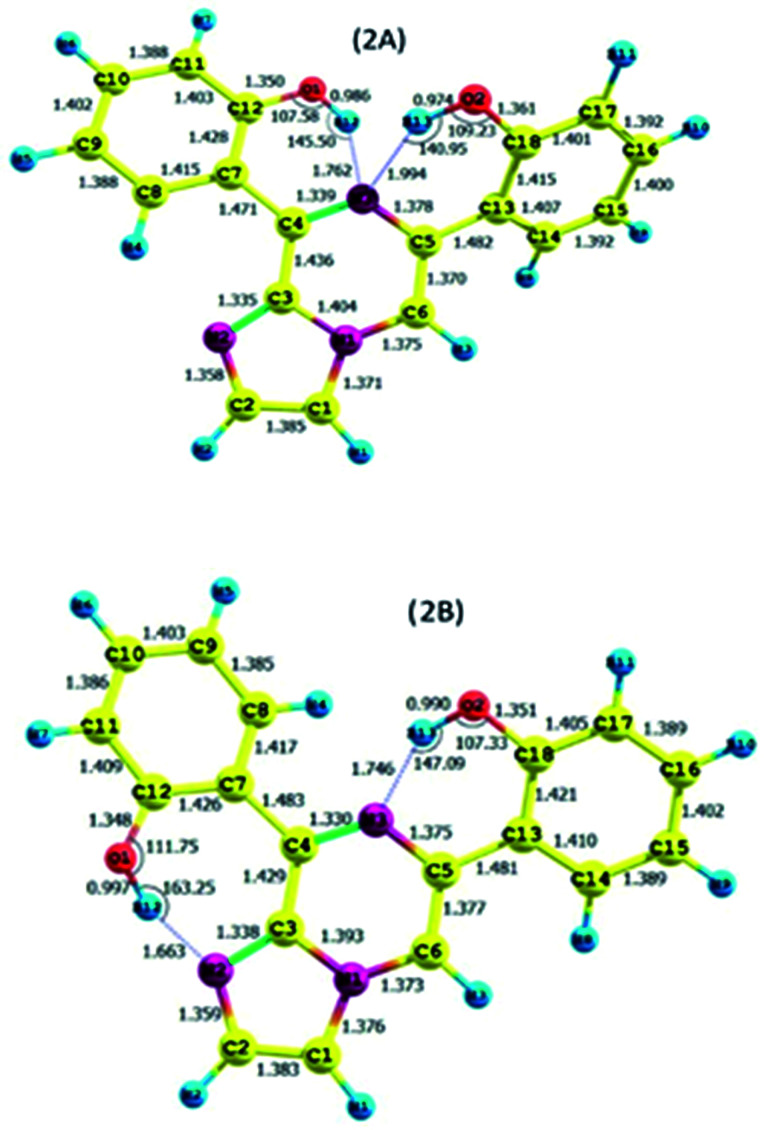
The optimized structures for compound 2.

As discussed above, the isomeric forms of compound 1 were found to have 1A and 1B geometry. Subsequently, the separated isomers were reacted with another boronic acid and this resulted in two new compounds. Therefore, the conformers that resulted from the second substitution could have the possible structures shown in Scheme 3. Conformer 1A, stabilized by six-membered hydrogen bonding, could further result in either form 2A or 2B. Therefore, compound 2 was first optimized based on conformer 2A using the same protocol as for compound 1. The optimized geometry was confirmed by it having no imaginary frequencies. Furthermore, the optimized structure of conformer 2A was analysed through potential energy surface analysis for a dual dihedral scan of two phenolic moieties with the imidazo-[1,2-*a*]pyrazine moiety with variation from 0° to 180° at a step size of 20 degrees. The Boltzmann contributions of significant structures with a relative energy of 5 kcal mol^−1^ on the potential energy surface were considered for further analysis. Four conformers represented by 2A, 2B, 2C and 2D were found to be within the Boltzmann energy limit for a significant contribution (Fig. 7). The relative energy and Boltzmann distributions for all conformers at the minima have been calculated in Table 4. All the conformers were found to be stabilized by hydrogen bonding, established by the interaction distance primarily. Conformer 2B was found to be the most stable with the highest contribution of 91.9%, followed by conformer 2A with an ∼6.7% contribution. On the other hand, 2C and 2D have negligible Boltzmann distributions. The molecular structures with adequate Boltzmann distributions have been optimized and analyzed by frequency calculations. The optimized structures for the conformers are shown in Fig. 8 along with their characteristic structural parameters, such as bond lengths and bond angles. The stabilization of the conformers has been discussed on the basis of hydrogen bonding.

**Table 4:** The relative free energy of the optimized conformations along with the Boltzmann distributions for compound 2

Conformer	Δ*E* (kcal mol^−1^)	Boltzmann distribution (%)
2A	1.530	6.96
2B	0	91.91
2C	18.508	0
2D	2.627	1.09

#### 3.6.1. Conformer 2A

Conformer 2A has an ∼7% Boltzmann contribution, stabilized by two six-membered hydrogen bonds, which was further quantified by the relative charges, and hydrogen bonding distances.

The calculated charges on the H12 [0.522 (NBO)], O1 [−0.699 (NBO)], H13 [0.519 (NBO)], O2 [−0.677 (NBO)] and N3 [−0.567 (NBO)] atoms establish N⋯H–O intramolecular dipolar interactions between a phenolic hydrogen and the pyrazine nitrogen of the imidazole moiety. The calculated non-bonded distance between the phenolic hydrogen (H12, H13) and pyrazine nitrogen (N3) [*d* (N3⋯H12) = 1.762 Å, and *d* (N3⋯H13) = 1.994 Å] has been found to be less than the sum of the van der Waals radii of hydrogen and nitrogen. The smaller distance and columbic interaction with opposite charges on H12, H13 and N3 support six-membered intramolecular hydrogen bonding.

#### 3.6.2. Conformer 2B

Conformer 2B has an ∼92% Boltzmann contribution, stabilized by six-membered and seven-membered hydrogen bonding, which is further quantified by the relative charges, and hydrogen bonding distances.

The calculated charges on the H12 [0.525 (NBO)], O1 [−0.710 (NBO)], N2 [−0.512 (NBO)], H13 [0.523 (NBO)], O2 [−0.722 (NBO)] and N1 [−0.540 (NBO)] atoms establish N⋯H–O intramolecular dipolar interactions between a phenolic hydrogen and the pyrazine/pyrrole nitrogen of the imidazole moiety.

The calculated non-bonded distances between the phenolic hydrogen (H12, H13) and pyrazine/pyrrole nitrogen (N3/N2) [*d* (N2⋯H12) = 1.663 Å, and *d* (N3⋯H13) = 1.764 Å] were found to be less than the sum of the van der Waals radii of hydrogen and nitrogen. The smaller distance and columbic interaction with opposite charges on H12, H13 and N3 support six-membered intramolecular hydrogen bonding.

Additionally, both conformers 2A and 2B were free from interelectronic repulsion. Furthermore, the nature of the hydrogen bonding interaction was estimated by QTAIM calculations using the Multiwfn program.

#### 3.6.3. Topology analysis

**Fig. 9:**
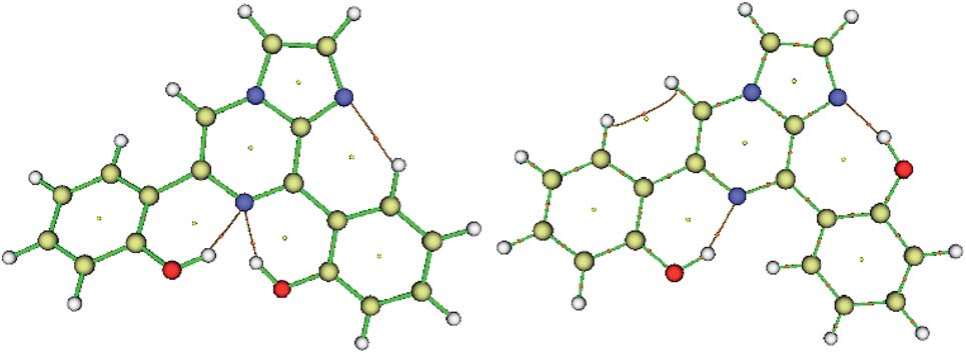
Molecular graphs for conformers 2A and 2B; the red dots denote the bond critical points along the bond paths.

The nature of the intramolecular hydrogen bonds existing within the key conformers 2A and 2B was established by means of Bader's QTAIM. Both conformers were stabilized by intramolecular hydrogen bonding and recognized by Popelier's criteria of hydrogen bonding. The topological parameters for hydrogen bonding are summarized in Table 5. It is found that in conformer 2A, N⋯H–O interactions of moderate strength with partial covalent character (∇^2^*ρ*(*r*_c_) > 0 and *H*(*r*) < 0) make the largest stabilization contribution of −6.18 and −11.1 kcal mol^−1^, while weak electrostatic ArH⋯N interactions (∇^2^*ρ*(*r*_c_) > 0 and *H*(*r*) > 0) make a stabilization contribution of −3.45 kcal mol^−1^. On the other hand, for the key conformer 2B, which demonstrated six-membered (6HB) and seven-membered (7HB) N⋯H–O and Ar–H⋯H–Ar interactions, it is ascertained that the N⋯H–O (6HB, 7HB) interactions contribute the majority of the stabilization with contributions of −11.42 and −13.71 kcal mol^−1^, while the Ar–H⋯H–Ar interaction^47^ makes a stabilization contribution of −1.76 kcal mol^−1^. The N⋯H–O (6HB, 7HB) interactions (∇^2^*ρ*(*r*_c_) > 0 and *H*(*r*) < 0) have moderate strength with partial covalent character, while the Ar–H⋯H–Ar interaction (∇^2^*ρ*(*r*_c_) > 0 and *H*(*r*) > 0) exhibits a weak electrostatic nature. The Laplacian of the electron density ∇^2^*ρ*(*r*_c_) and stabilization energy for the six-membered N⋯H–O hydrogen bonds have nearly the same value for both conformers 2A and 2B. However, the Laplacian of the electron density ∇^2^*ρ*(*r*_c_) and stabilization energy for the seven-membered N⋯H–O hydrogen bonds have a significantly higher value for conformer 2B relative to the six-membered N⋯H–O hydrogen bonds for conformer 2A, which resulted in the higher Boltzmann contribution for conformer 2B. The bond critical points and bond paths for both forms are shown in Fig. 9.

**Table 5:** Topology parameters, including electron density(*ρ*), Laplacian electron density (∇^2^*ρ*), potential energy density [*V*(*r*)], total energy density [*H*(*r*)], and hydrogen bonding energy [*E*_HB_ (kcal mol^−1^)] at the bond critical point of the non-covalent interactions (D⋯HA) for conformers 2A and 2B at the B3LYP/6-31++G** level

Compound	Interaction	BCP	*d* (Å)	∠D⋯HA	∇^2^*ρ*	*V*(*r*)	*G*(*r*)	*H*(*r*)	*E*_HB_
2A									
	N⋯HO	(3, −1)	1.994	140.95	0.0742	−0.0197	0.0191	−0.0006	−06.18
	N⋯HO	(3, −1)	1.762	145.50	0.1151	−0.0354	0.0321	−0.0033	−11.10
	ArH⋯N	(3, −1)	2.265	123.12	0.0577	−0.0110	0.0127	0.0017	−3.45

2B									
	N⋯HO (7HB)	(3,−1)	1.663	163.25	0.1240	−0.0437	0.0374	−0.0064	−13.71
	N⋯HO (6HB)	(3, −1)	1.746	147.09	0.1165	−0.0364	0.0327	−0.0036	−11.42
	ArH⋯HAr	(3, −1)	2.144		0.0471	−0.0056	0.0087	0.0030	−1.76

#### 3.6.4. MBO and LBO analysis for compound 2

Furthermore, the Mayer bond order (MBO) and Laplacian bond order (LBO) were analyzed for the stable conformers 2A and 2B (Table 6). The MBO and LBO indexes for the O–H bond have values less than 1, clearly indicating the polarization of the bond, which further allows the hydrogen atom to interact with the nitrogen atom through hydrogen bonding, thus validating the intramolecular hydrogen bonding. The low value of the LBO and MBO indexes for conformer 2B for O–H bonds indicated the high bond polarization compared to conformer 2A. Additionally, the polarization of both O–H bonds was reasonably supported by them having almost the same LBO and MBO indexes in conformer 2B, while the difference in the indexes for the two O–H bonds for conformer 2A indicated the difference in the strength of the bonds.

**Table 6:** Effective bond order for conformers 2A and 2B

Bond	MBO at 6-31G**		LBO at 6-31++G**	
	2A	2B	2A	2B
N2–H12	—	0.1702	—	—
N3–H12	0.1268	—	—	—
N3–H13	0.0764	0.1317	—	—
C12–O1	1.0336	1.0451	0.5755	0.5775
C18–O2	0.9226	1.0342	0.5455	0.5775
O1–H12	0.7663	0.7261	0.5433	0.4993
O2–H13	0.8114	0.7565	0.6042	0.5364

#### 3.6.5. Frequency analysis

As discussed earlier, the free O–H stretching vibration occurs between 3500–3700 cm^−1^ and the hydrogen bonded O–H stretching vibration is usually observed in the range of 3200–3600 cm^−1^. The simulated FTIR spectrum for conformer 2A has broad signals for O–H bonds at 3695.05, and 3391.24 cm^−1^, while conformer 2B has O–H signals at 3159.33 and 3233.54 cm^−1^. As discussed above, conformer 2A has two O–H bonds of different strengths, and consequently the calculated FTIR signals were found at a higher wavenumber with a significant difference of ∼300 cm^−1^. The experimentally observed FTIR signals for conformer 2A were found at 3100, and 3030 cm^−1^ (Fig. S15†). On the other hand, conformer 2B has O–H bonds of almost the same strength and high stabilization; thus, the calculated FTIR signals were found at lower wavenumbers with a small difference of ∼70 cm^−1^. Likewise, the observed FTIR signals for conformer 2B were at 3000, and 2930 cm^−1^ (Fig. S20†). The observed and calculated FTIR signals for the O–H bonds in both conformers were in correlation with their strength and the polarization of the bonds.

#### 3.6.6. NMR

**Fig. 10:**
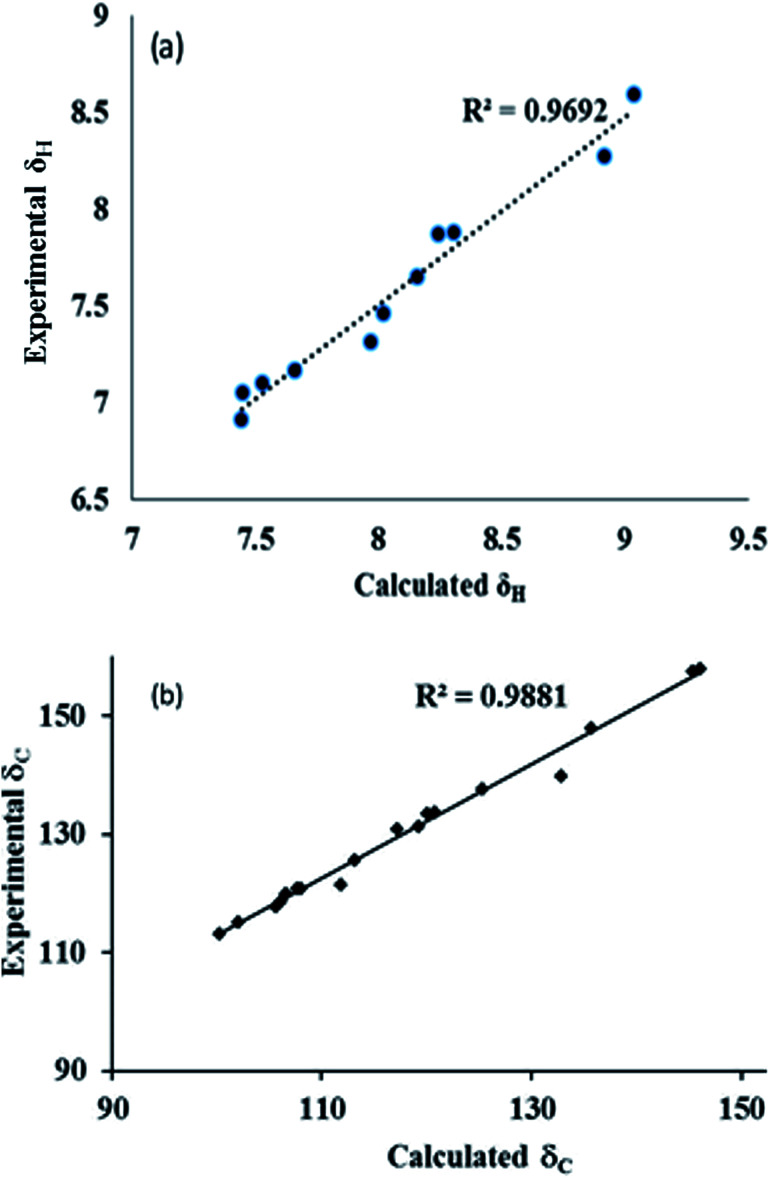
Experimental and theoretical correlation of (a) ^1^H and (b) ^13^C NMR for conformer 2B.

The ^1^H and ^13^C NMR shielding tensors were calculated for conformers 2A and 2B using the GIAO method on the optimized structures, and the effect of the solvent is included using the IEFPCM model provided in Gaussian 03 W. The assessment of the computed and experimental ^13^C NMR results revealed good correlation for both conformers 2A and 2B. The correlation value for ^13^C NMR was found to be *R*^2^ = 0.97 and 0.99 for conformer 2A and 2B, respectively. Furthermore, the correlation value of the computed and experimental ^1^H NMR results for conformer 2B was *R*^2^ = 0.97. However, the correlation of the ^1^H NMR results for conformer 2A was *R*^2^ = 0.90. The poor correlation could be due to deviation in the OH signals. The correlation of the ^1^H NMR and ^13^C NMR results for conformer 2B is shown in Fig. 10. Similar to compounds 1A and 1B, it has been observed that the hydrogen atoms involved in hydrogen bonding resonate distinctly for 2A and 2B. The proton signals of C6′ and C6′′ have been effectively influenced depending upon the strength of the hydrogen bonding involved. Compound 2A resonates between 8.59 and 6.97 ppm, where the signal resulting from C6′ is observed at 8.59 ppm. On the other hand, compound 2B resonates between 10.7 and 6.82 ppm, where the aromatic proton signal involved in hydrogen bonding occurs at 10.7 ppm. Additionally, the ^1^H NMR results of both compounds 2A and 2B also differ in the aromatic proton signals (Table S3†). A comparison of the NMR results for conformer 2A is shown in Fig. S16.†

#### 3.6.7. Absorption properties

The vertical Franck–Condon excitations were calculated for the optimized structures of conformers 2A and 2B, using TD-DFT, and further compared with the experimental absorption spectra. The obtained absorption values for conformer 2A at 380, 360 and 265 nm have a major contribution from three Franck–Condon excited states [(S_0_–S_1_), (S_0_–S_5_) and (S_0_–S_6_)], respectively. On the other hand, the experimental absorption peaks for conformer 2B were observed at 375, 315, and 260 nm. The experimentally observed transitions were found to be due to [(S_0_–S_1_), (S_0_–S_2_), and (S_0_–S_6_)] Franck–Condon transitions, respectively. The calculated and experimental absorption spectral data are tabulated in Table S4.† The different contributing molecular orbitals are shown in Fig. S22.†

Thus, the differences in the signals for the NMR, FTIR, and absorption peaks for the two compounds and their correlation with theoretical calculations clearly predict the specific orientation of the rotamers.

## 4. Conclusions

In summary, we have successfully synthesized a series of imidazo-[1,2-*a*]pyrazines, possessing different rotameric conformations, which were further purified by column chromatography. Theoretical investigation of the accurate structure of the different rotameric forms was done through PES and hydrogen bond strength analysis. The obtained rotameric forms were found to be stabilized by intramolecular hydrogen bond locking, which was further established by QTAIM analysis. The Laplacian and Mayer bond order (LBO/MBO) revealed that differences in bond strength for the different conformations, and thus distinctions in the vibrational frequency and NMR signals, assisted in the recognition of the accurate structure for the different conformations.

## Conflicts of interest

There are no conflicts of interest to declare.

## Supplementary Material

RA-008-C7RA13617J-s001
