# Supplementary material for: Investigation of rotameric conformations of substituted imidazo-[1,2-a]pyrazine: experimental and theoretical approaches
Source: RSC Adv. 2018 Mar 7;8(18):9707–17. doi: 10.1039/c7ra13617j (PMC9078725; doi:10.1039/c7ra13617j)
Supplement: RA-008-C7RA13617J-s001 [file RA-008-C7RA13617J-s001.pdf]

Electronic Supplementary Material (ESI) for RSC Advances.
This journal is © The Royal Society of Chemistry 2018

Supporting Information for

Investigation of rotameric conformations of substituted imidazo- [1, 2-a] pyrazine: experimental and theoretical approaches

Gulshan Kumar, Richa Goel, Kamaldeep Paul and Vijay Luxami*

School of Chemistry and Biochemistry, Thapar University, Patiala, Punjab, India

E-mail: vluxami@thapar.edu

	Content	Page No.
**Figure S1**	^{1}H NMR spectra of Compound **1A**	2
**Figure S2**	^{13}C NMR spectra of Compound **1A**	2
**Figure S3**	Mass spectra of compound **1A**	3
**Figure S4**	FTIR spectra of compound **1A**	3
**Figure S5**	^{1}H NMR spectra of Compound **1B**	4
**Figure S6**	^{13}C NMR spectra of Compound **1B**	4
**Figure S7**	Mass spectra of compound **1B**	5
**Figure S8**	FTIR spectra of compound **1B**	5
**Figure S9**	Experimental and theoretical ^{1}H NMR (left) and ^{13}C NMR (right) correlation for conformer **1B**	6
**Table S1**.	Experimental ^{1}H NMR signals for conformer **1A** and **1B**	6
**Figure S10**	The absorption spectra of different compound **1A** and **1B** in CH_3CN.	7
**Table S2**	The calculated absorption value for conformer **1A** and **1B** in gas and CH_3CN solvent phase at B3LYP/6-31++G** level of theory, symmetry of frontier orbital and % contributions of Molecular orbitals	7
**Figure S11**	Frontier Molecular orbital for conformer **1B**	8
**Figure S12**	^{1}H NMR spectra of Compound **2A**	9
**Figure S13**	^{13}C NMR spectra of Compound **2A**	9
**Figure S14**	Mass spectra of compound **2A**	10
**Figure S15**	FTIR Spectra of compound **2A**	11
**Figure S16**	Experimental and theoretical ^{1}H NMR (left) and ^{13}C NMR (right) correlation for conformer **2A**	11
**Figure S17**	^{1}H NMR spectra of Compound **2B**	12
**Figure S18**	^{13}C NMR spectra of Compound **2B**	12
**Figure S19**	Mass spectra of compound **2B**	13
**Figure S20**	FTIR spectra of compound **2B**	13
**Table S3**	Experimental ^{1}H NMR signals for conformer **2A** and **2B**	14
**Figure S21**	The absorption spectra of different conformers **2A** and **2B** in CH_3CN.	14
**Table S4**	The calculated absorption value for conformer **2A** and **2B** in gas and CH_3CN solvent phase at B3LYP/6-31++G** level of theory, symmetry of frontier orbital and % contributions of Molecular orbitals	15
**Figure S22**	Frontier Molecular orbital for conformer **2B**	17

Figure S23 Frontier Molecular orbital for conformer **2A** 18

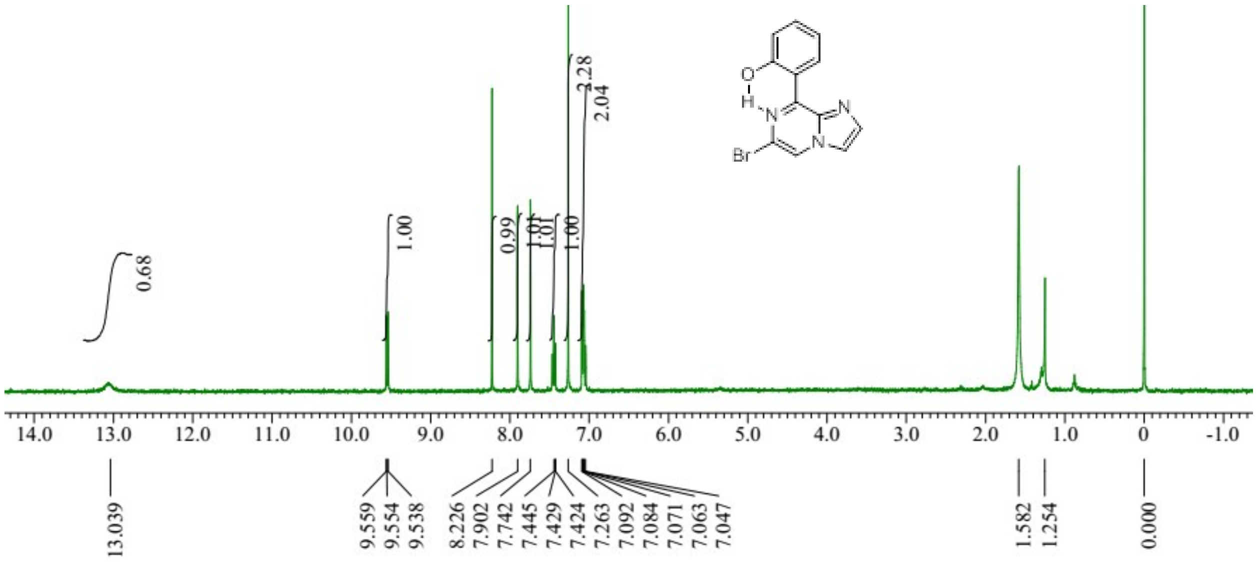

Figure S1: ^{1}H NMR spectra of Compound **1A**

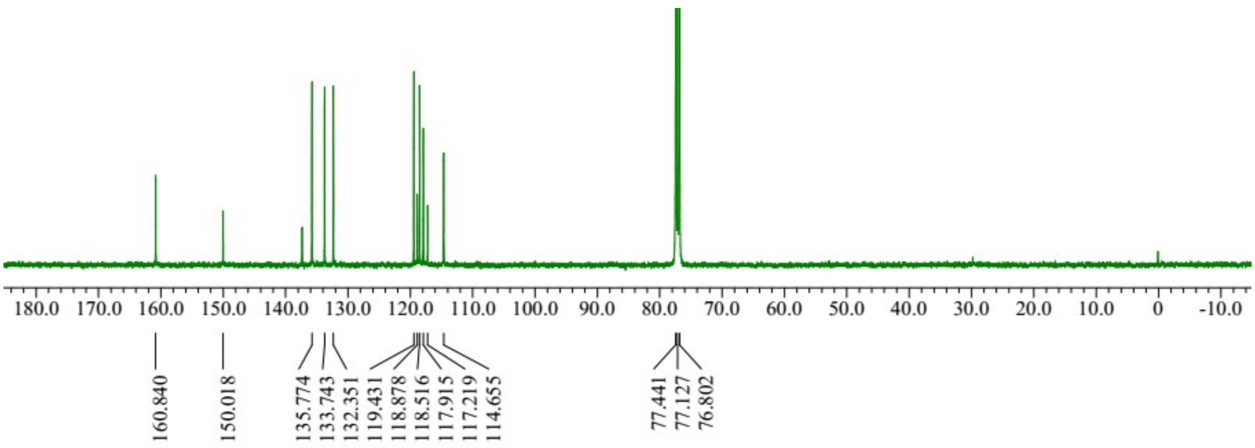

Figure S2: ^{13}C NMR spectra of Compound **1A**

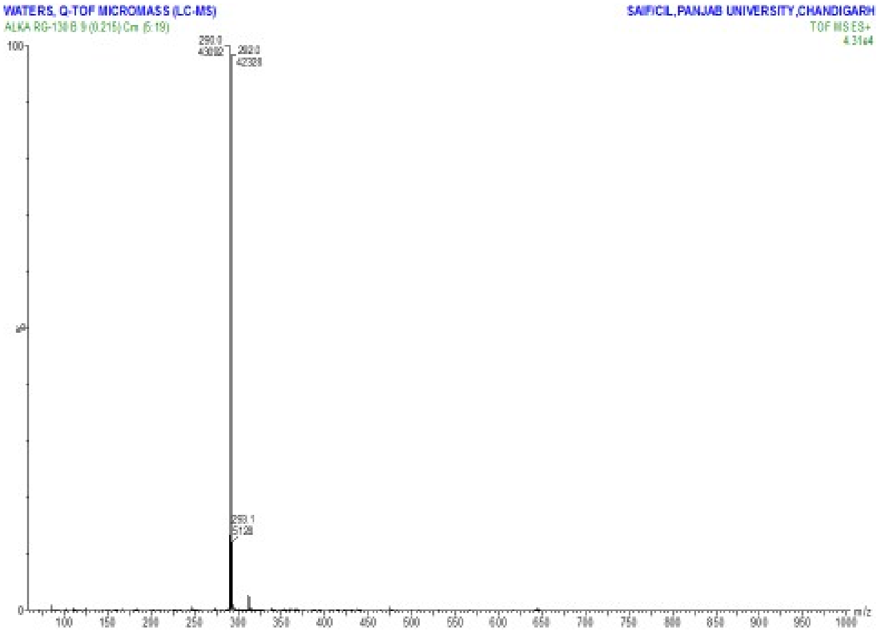

Figure S3: Mass spectra of compound **1A**

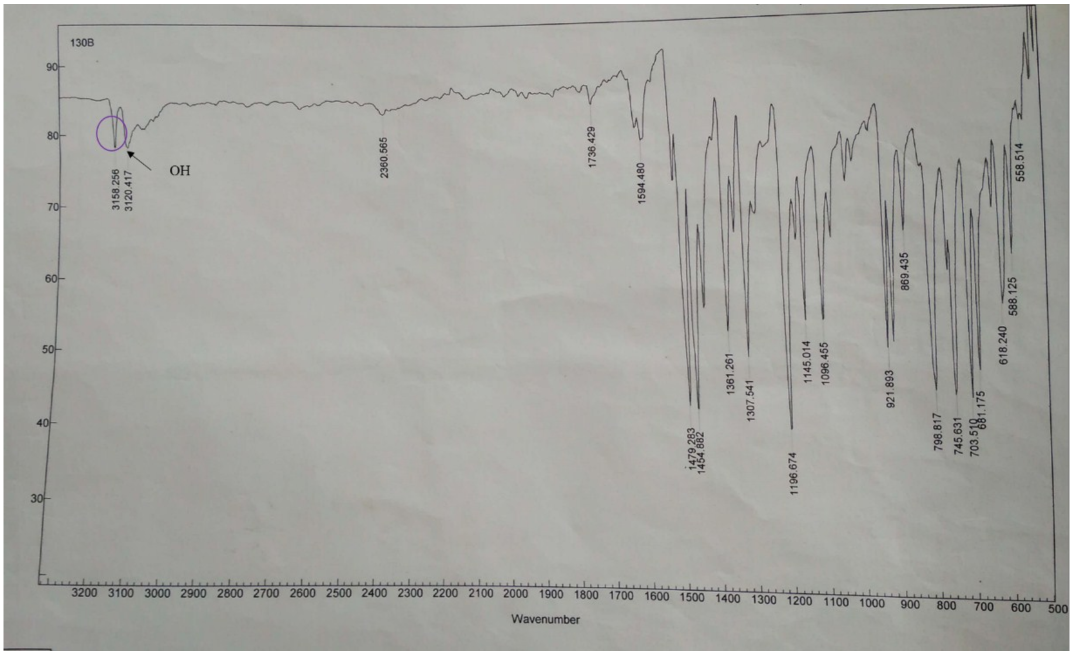

Figure S4: FTIR Spectra of compound **1A**

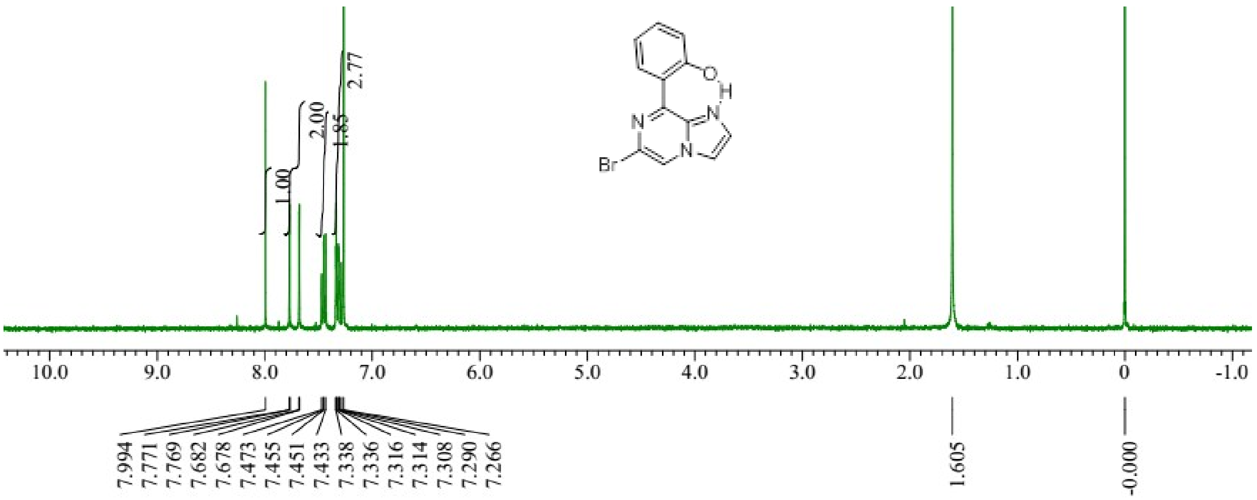

2.77
2.00
1.85
1.00
Br
N
N
N
O
H
10.0
9.0
8.0
7.0
6.0
5.0
4.0
3.0
2.0
1.0
0
-1.0
7.994
7.771
7.769
7.682
7.678
7.473
7.455
7.451
7.433
7.338
7.336
7.316
7.314
7.308
7.290
7.266
1.605
-0.000

Figure S5: ^{1}H NMR spectra of Compound **1B**

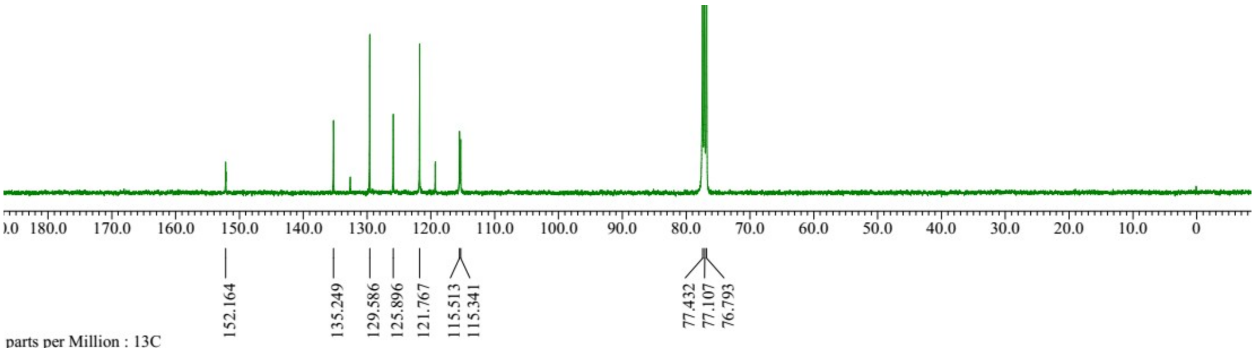

Figure S6: ^{13}C NMR spectra of Compound **1B**

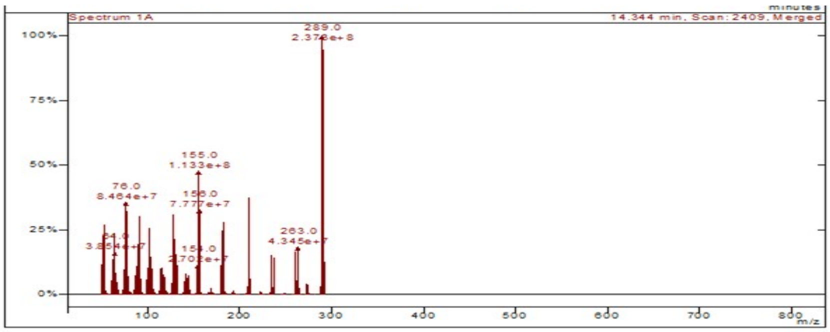

Figure S7: Mass spectra of compound **1B**

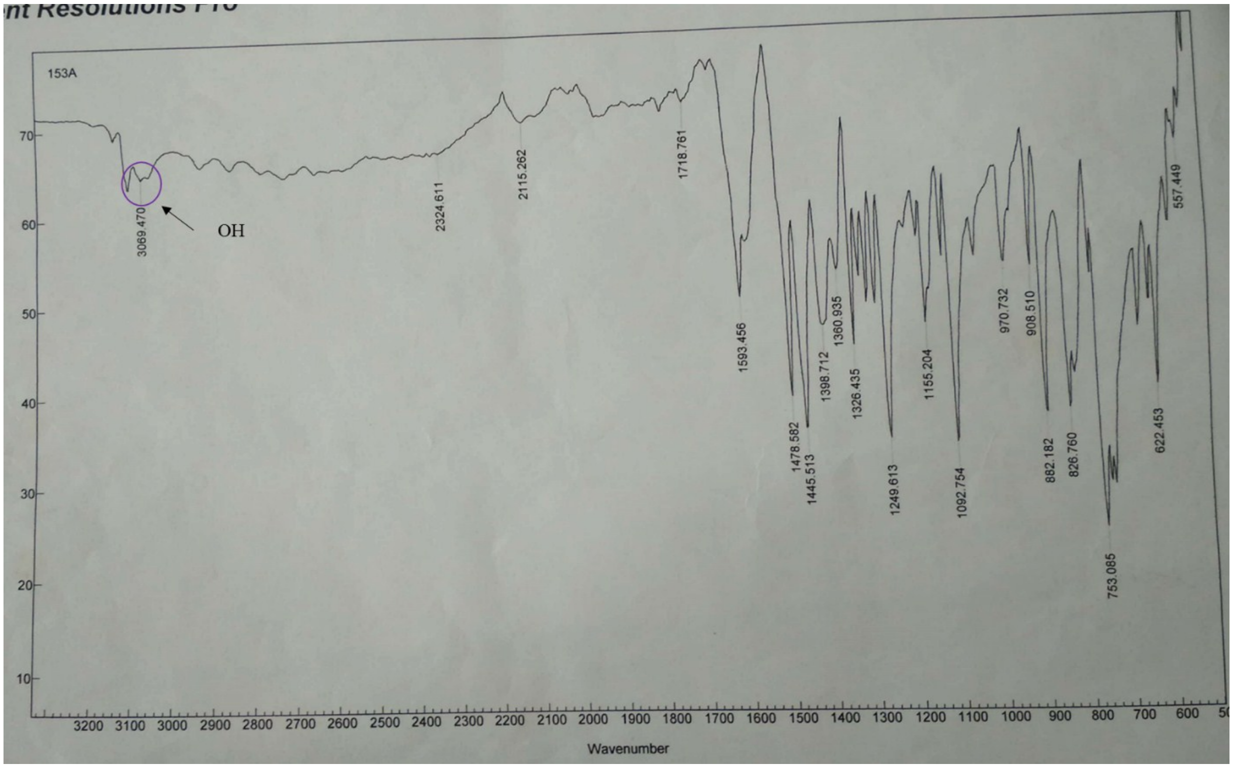

Figure S8: FTIR Spectra of compound **1B**

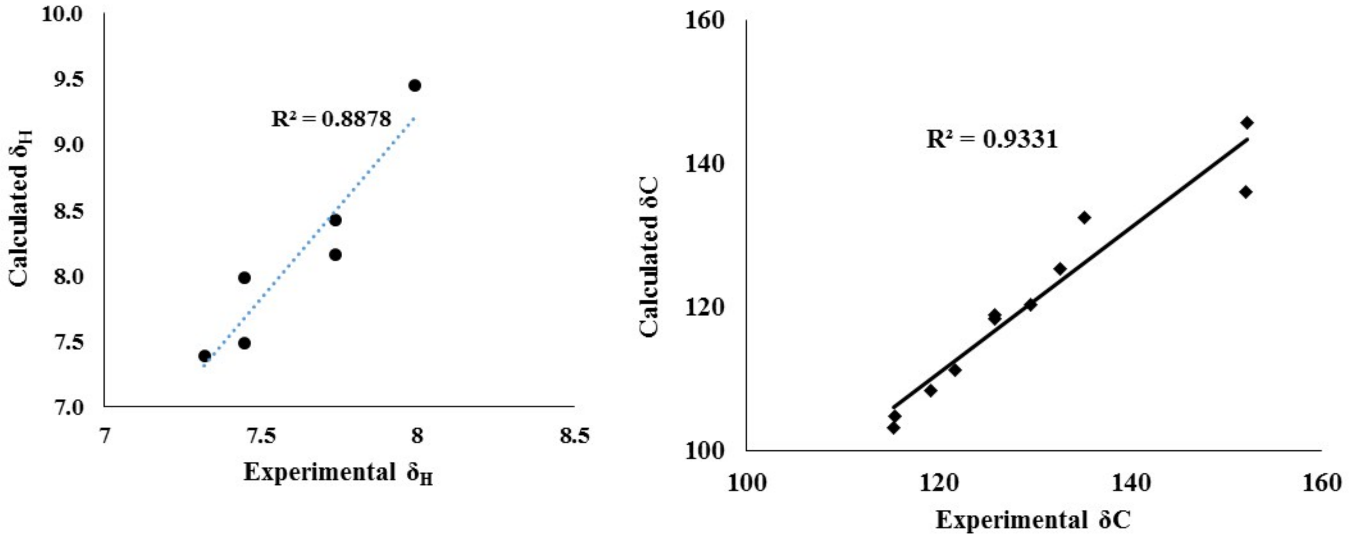

Figure S9: Experimental and theoretical ^{1}H NMR (left) and ^{13}C NMR (right) correlation for conformer **1B**

**Table S1.** Experimental ^{1}H NMR signals for conformer **1A** and **1B**		
**H- position**	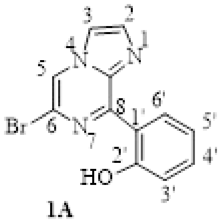 1A	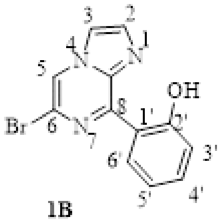 1B
	13.03	
6'	9.54	7.99
5	8.23	7.77
2	7.90	7.68
3	7.74	7.47
4'	7.45	7.43
3'	7.05	7.34
5'	7.09	7.29

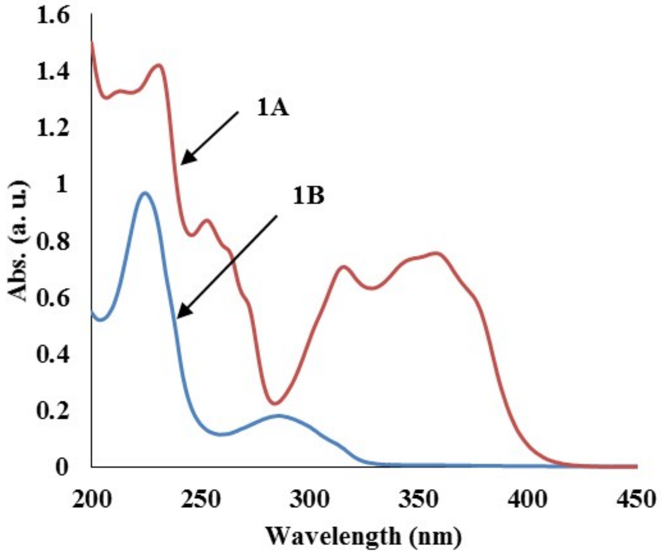

Figure S10: The absorption spectra of different compound **1A** and **1B** in CH_3CN.

Table S2: The calculated absorption value for conformer **1A** and **1B** in gas and CH_3CN solvent phase at B3LYP/6-31++G** level of theory, symmetry of frontier orbital and % contributions of Molecular orbitals

Excited State	λ_{ACN} (nm)	λ_{Gas} (nm)	λ_{Exp} (nm)	Osc. Strength	Symmetry	% Major Orbital Contribution	
				**1A**			
S_0–S_1	370.20	388.50	380,	0.2567	Singlet-A	H→L	94
S_0-S_2	329.15	326.29		0.1214	Singlet-A	H-1→L	89
S_0-S_3	311.03	309.84	361,	0.184	Singlet-A	H-2→L	86
S_0-S_4	274.96	281.59		0.0001	Singlet-A	H-4→L	96
S_0-S_5	270.69	281.16	318	0.155	Singlet-A	H-3→L	57
S_0-S_6	262.13	269.59		0.0688	Singlet-A	H→L+1	53
				**1B**			
S_0-S_1	381.54	402.60		0.2699	Singlet-A	H→L	94
S_0-S_2	333.23	336.02		0.1178	Singlet-A	H-1→L	94
S_0-S_3	300.16	300.28	290	0.1204	Singlet-A	H-2→L	81
S_0-S_4	280.89	288.76		0.0006	Singlet-A	H-4→L	95
S_0-S_5	278.85	288.73		0.1195	Singlet-A	H-3→L	44
S_0-S_6	265.55	274.51		0.0294	Singlet-A	H-3→L	43

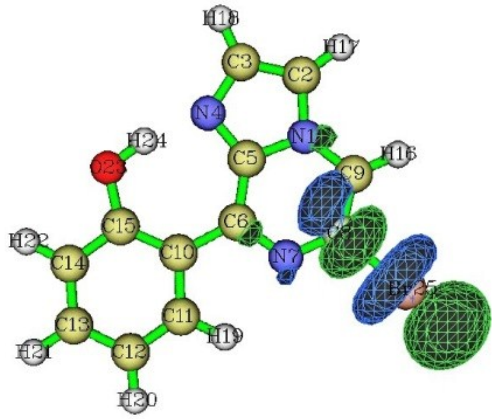

LUMO+2

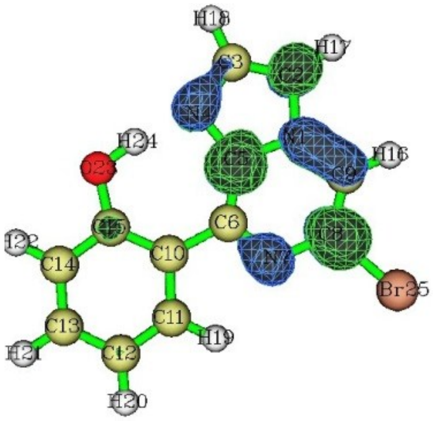

LUMO+1

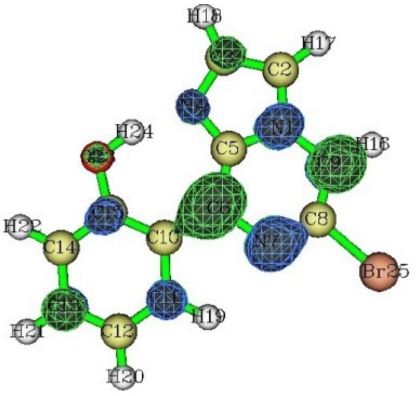

LUMO

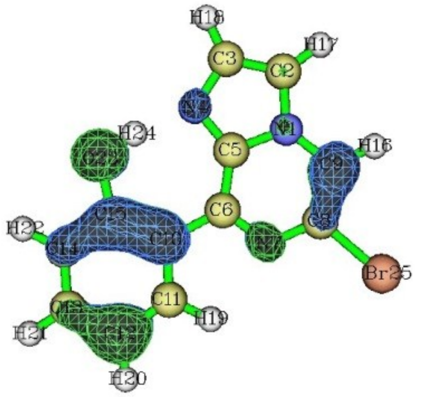

HOMO

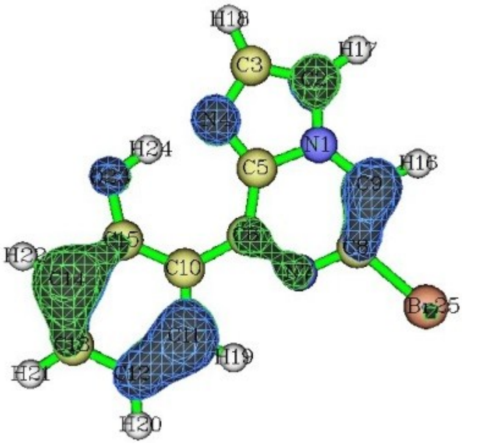

HOMO-1

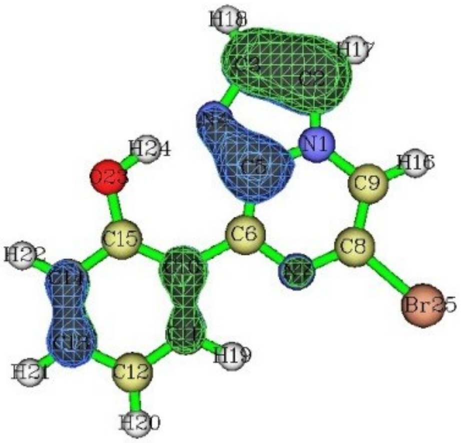

HOMO-2

Figure S11: Frontier Molecular orbital for conformer **1B**

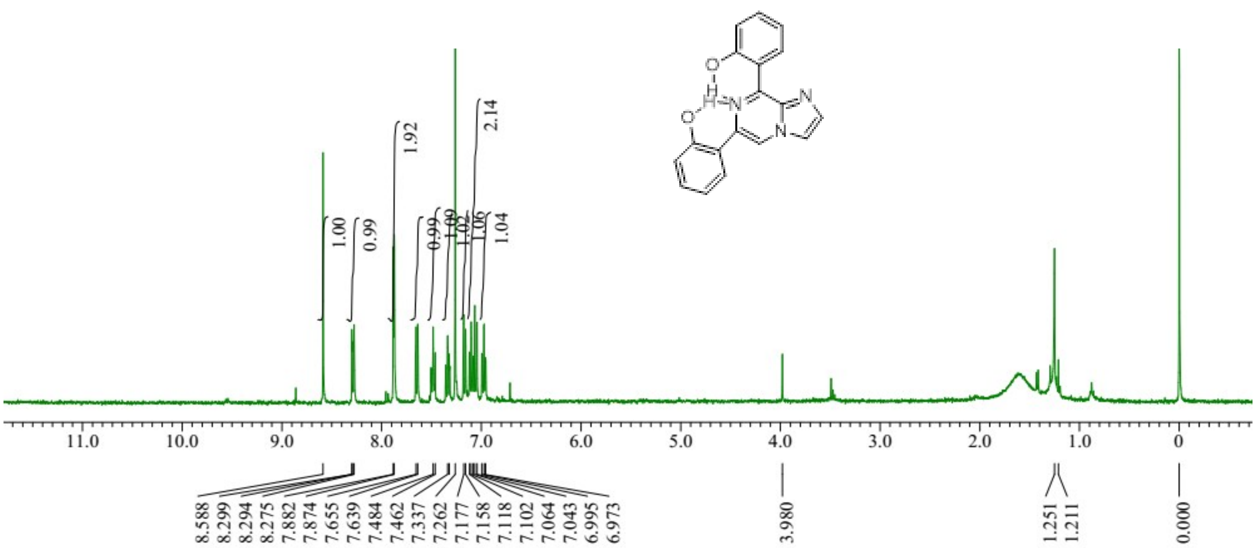
1.00
0.99
1.92
0.99
1.00
1.02
1.06
1.04
2.14
11.0
10.0
9.0
8.0
7.0
6.0
5.0
4.0
3.0
2.0
1.0
0
8.588
8.299
8.294
8.275
7.882
7.874
7.655
7.639
7.484
7.462
7.337
7.262
7.177
7.158
7.118
7.102
7.064
7.043
6.995
6.973
3.980
1.251
1.211
0.000

Figure S12: ^{1}H NMR spectra of Compound **2A**

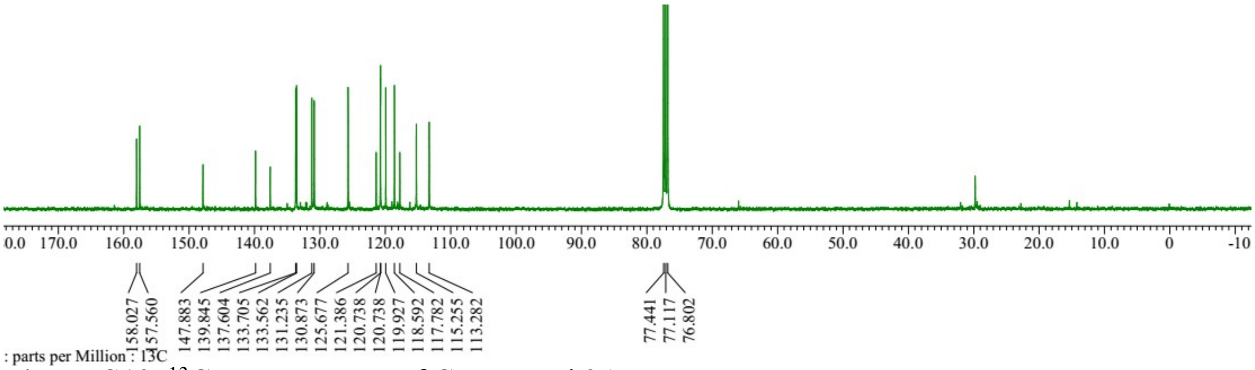

Figure S13: ^{13}C NMR spectra of Compound **2A**

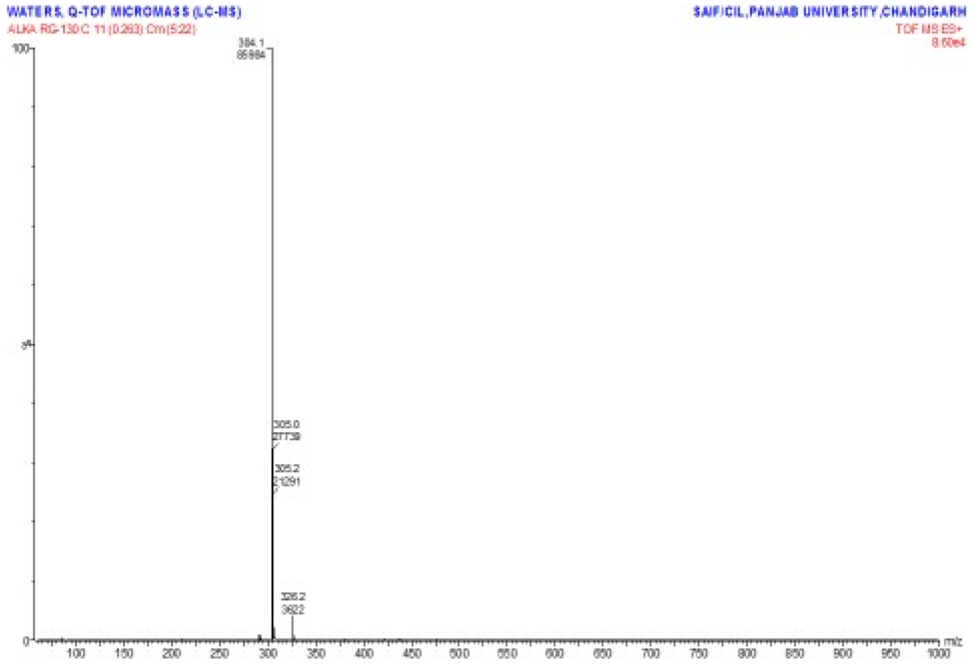

Figure S14: Mass spectra of compound **2A**

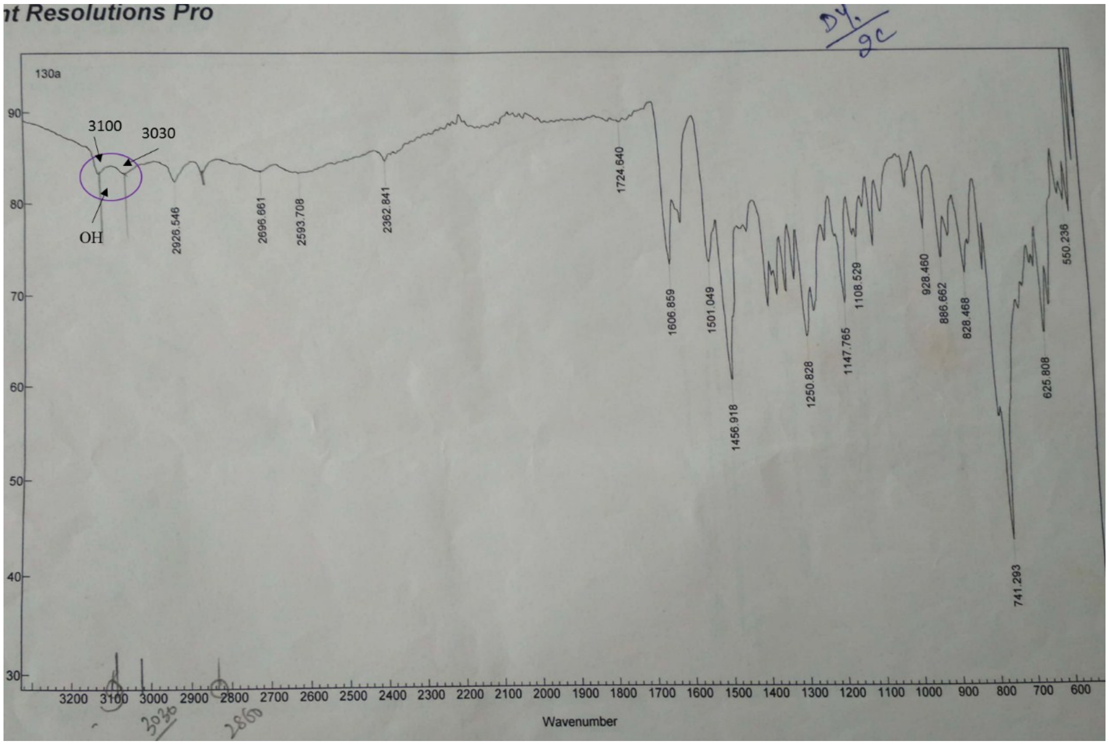

Figure S15: FTIR Spectra of compound **2A**

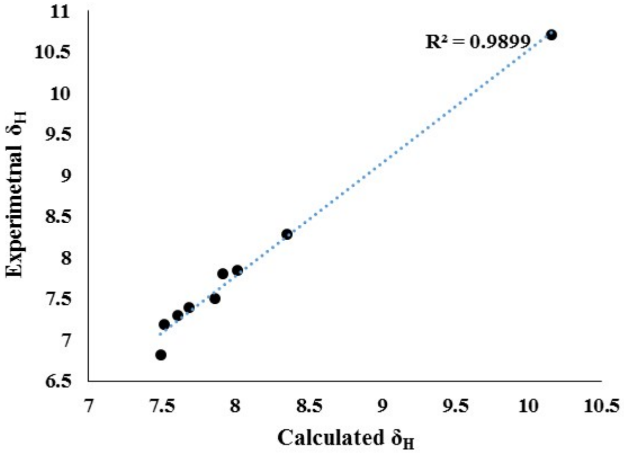

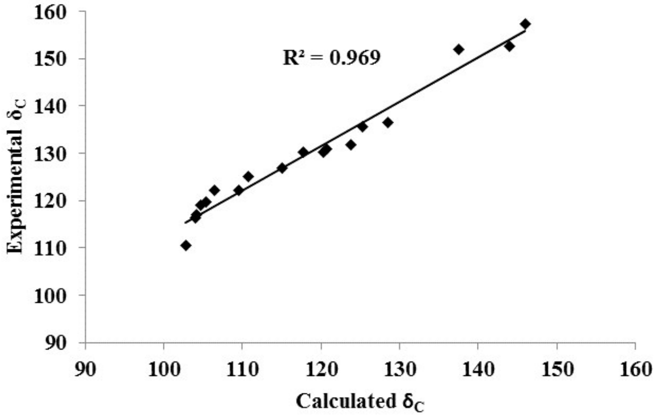

Figure S16: Experimental and theoretical ^{1}H NMR (left) and ^{13}C NMR (right) correlation for conformer **2A**

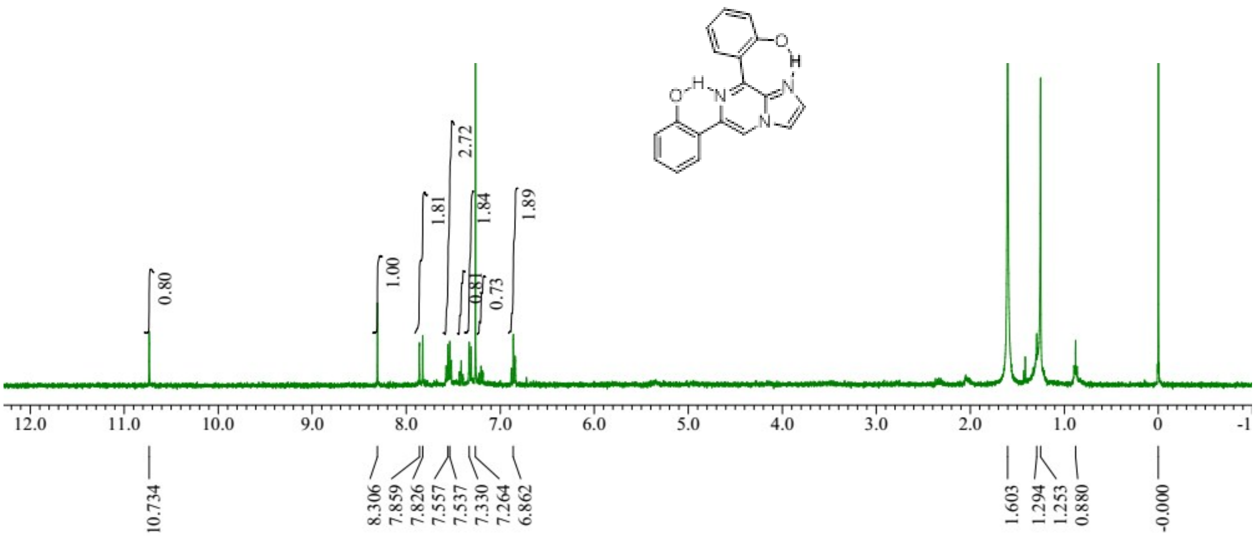

Figure S17: ^{1}H NMR spectra of Compound **2B**

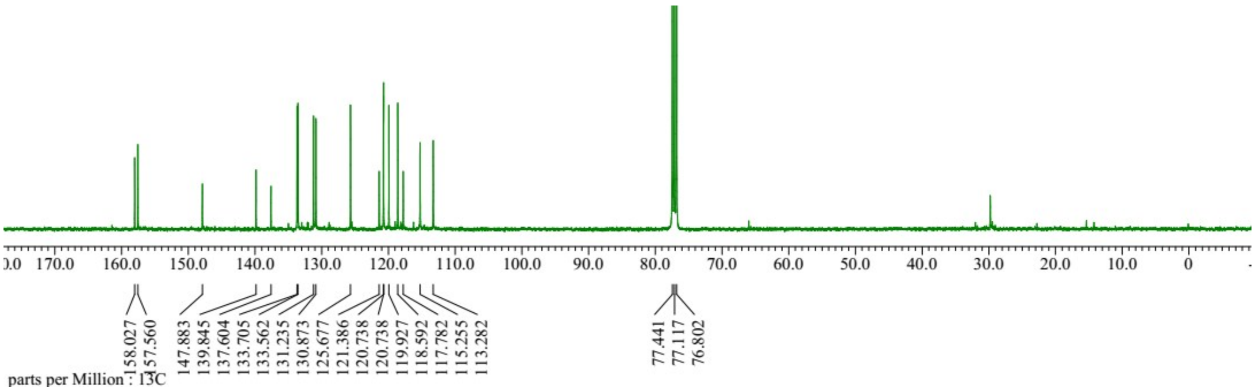

Figure S18: ^{13}C NMR spectra of Compound **2B**

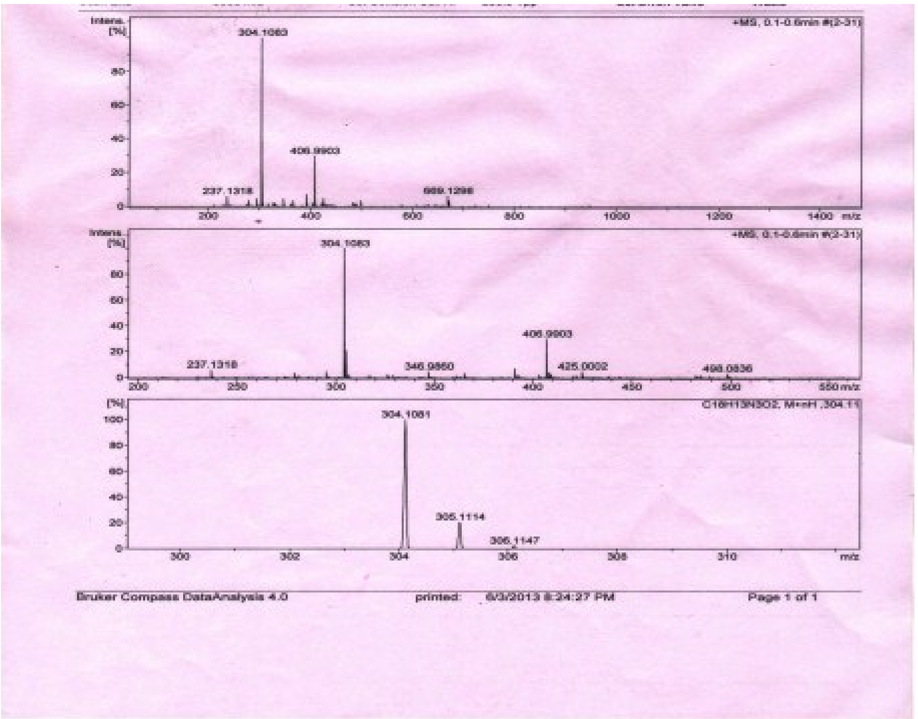

Figure S19: Mass spectra of compound **2B**

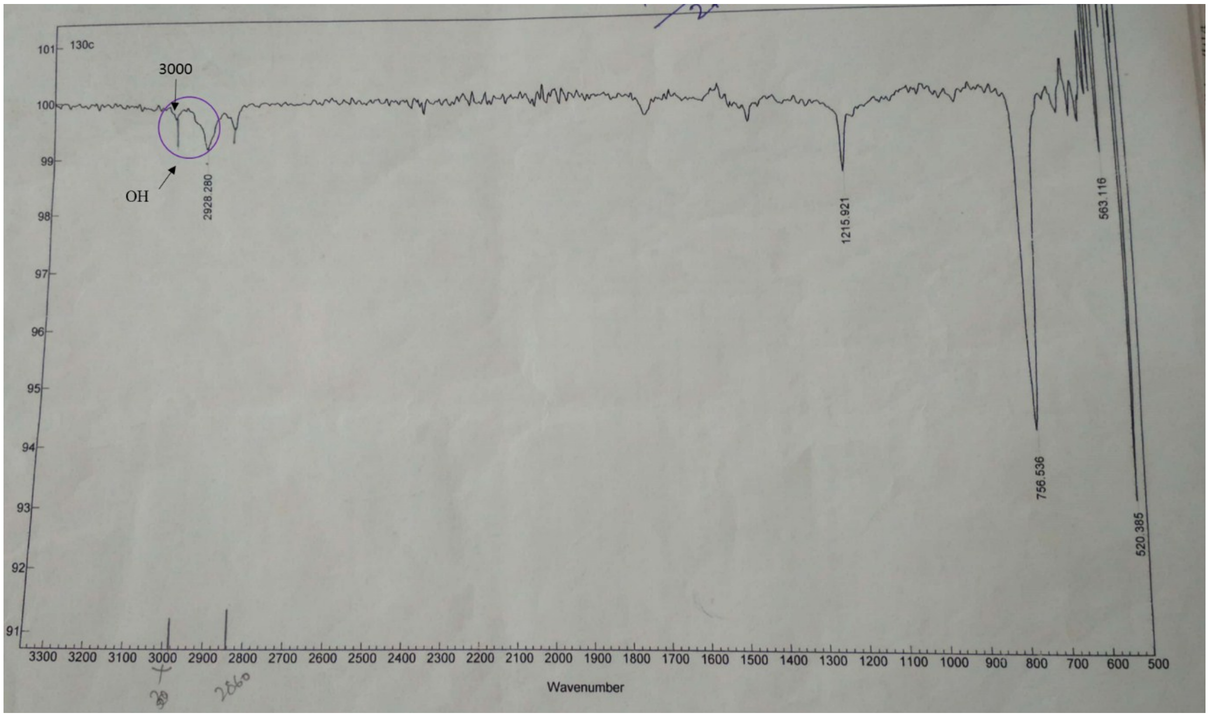

Figure S20: FTIR spectra of compound **2B**

Table S3. Experimental ^{1}H NMR signals for conformer **2A** and 2**B**

	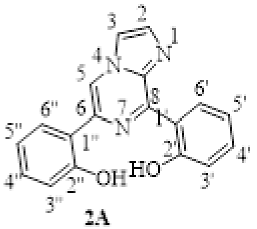 2A	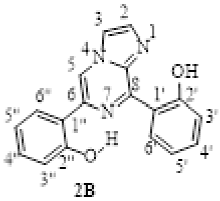 2B
	**2A**	**2B**
6'	8.59	10.7
5	8.29	7.84
2	7.88	7.8
3	7.65	7.5
4'	7.48	7.39
4"	7.33	7.29
6"	7.26	
3'	7.18	
3"	7.12	
5'	7.06	7.18
5"	6.97	6.82

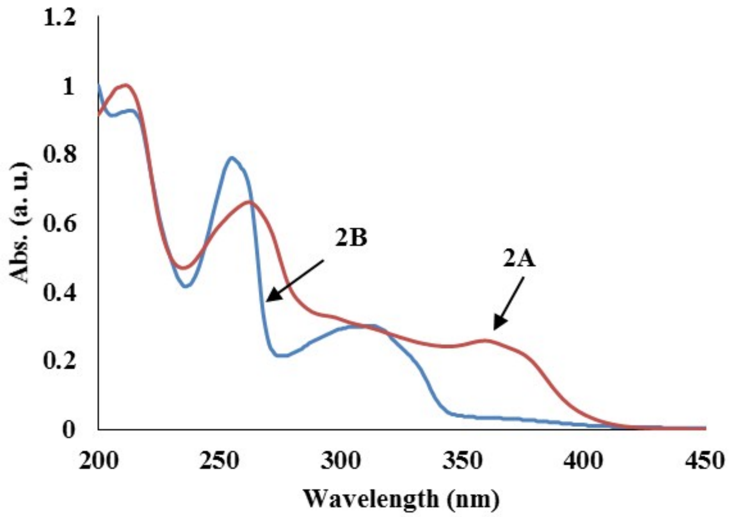

Figure S21: The absorption spectra of different conformers **2A** and **2B** in CH_3CN.

Table S4: The calculated absorption value for conformer **2A** and **2B** in gas and CH_3CN solvent phase at B3LYP/6-31++G** level of theory, symmetry of frontier orbital and % contributions of Molecular orbitals

Excitation S_0-S_n	λ_{Gas} (nm)	λ_{ACN} (nm)	λ_{Exp} (nm)	Osc. Strength	Symmetry	Major orbital contribs.	
**2A**							
1	387.15	377.15	380,	0.1640	Singlet-A	H→L	96%
2	375.78	357.40	360,	0.0624	Singlet-A	H-1→L	98%
3	326.38	325.97	265	0.0625	Singlet-A	H-2→L	89%
4	315.62	313.60		0.1080	Singlet-A	H-3→L	63%
5	297.45	290.59		0.4008	Singlet-A	H→L+1	55%
6	292.17	286.91		0.2020	Singlet-A	H-4→L	81%
**2B**							
1	406.33	391.19	375,	0.1003	Singlet-A	H→L	91%
2	389.98	362.46	315,	0.1141	Singlet-A	H-1→L	93%
3	335.74	329.86	260	0.073	Singlet-A	H-2→L	93%
4	321.73	317.88		0.1328	Singlet-A	H→L+1	80%
5	306.73	297.46		0.2859	Singlet-A	H-1→L+1	61%
6	301.69	292.03		0.2095	Singlet-A	H-3→L	59%

2A

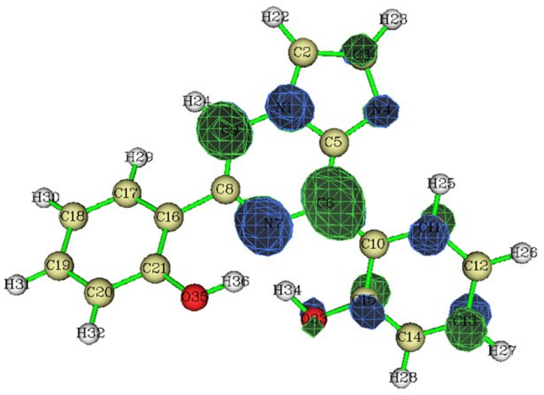

LUMO

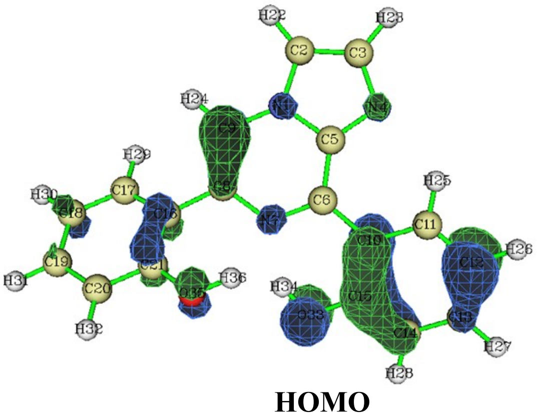

HOMO

2B

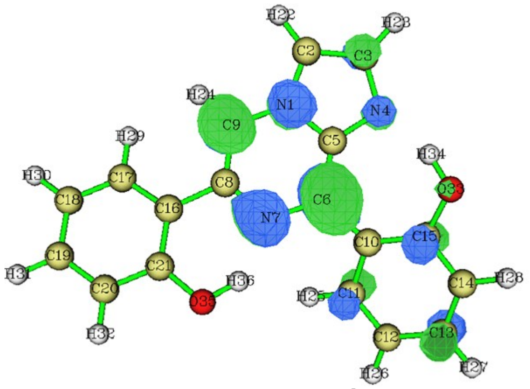

LUMO

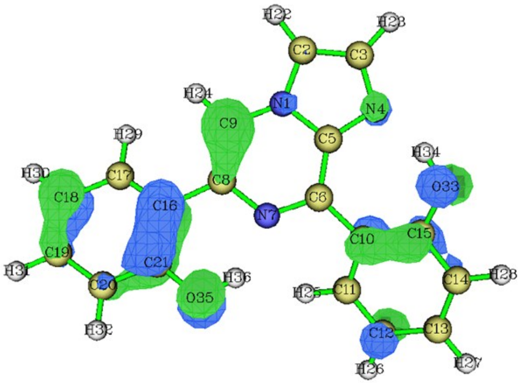

HOMO

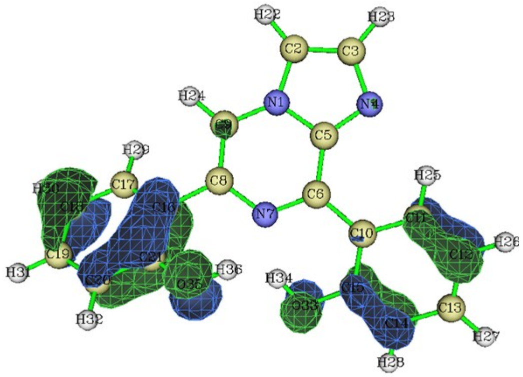

HOMO-1

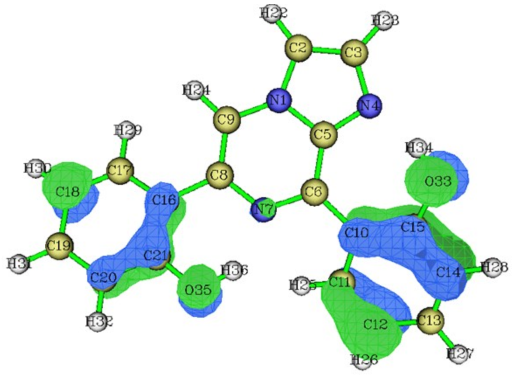

HOMO-1

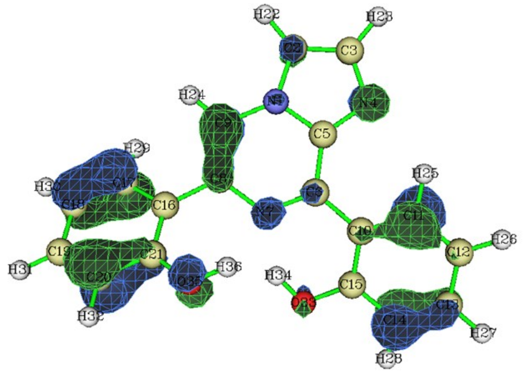

HOMO-2

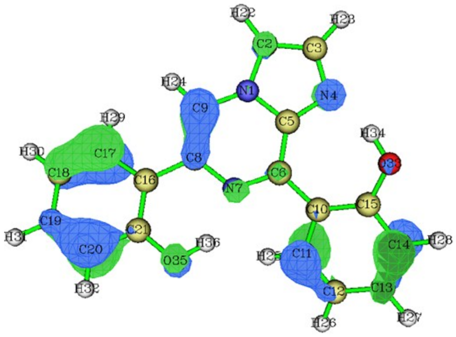

HOMO-2

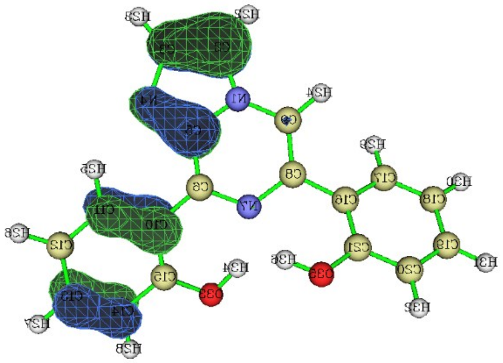

HOMO-3

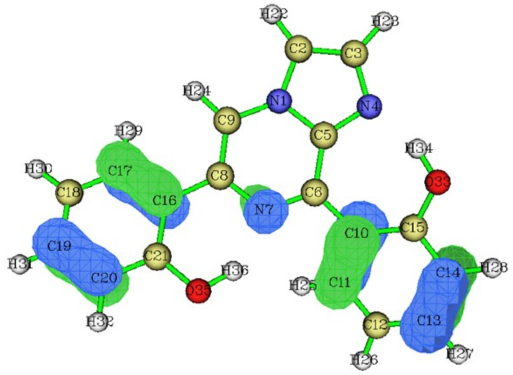

HOMO-3

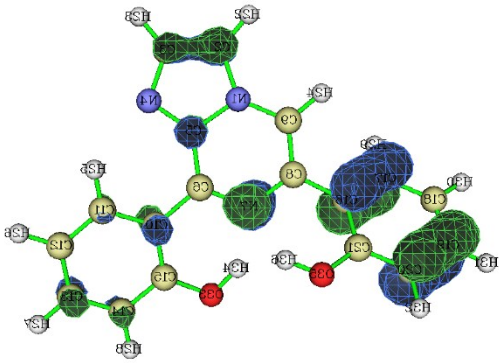

HOMO-4

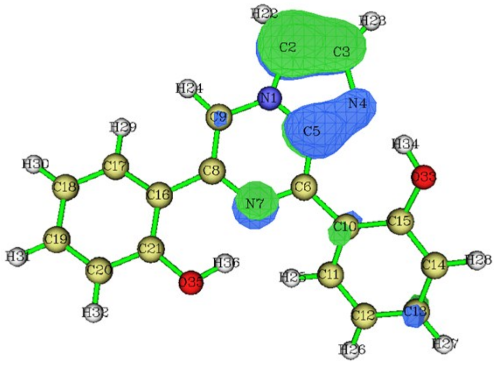

HOMO-4

Figure S22: Frontier Molecular orbital for conformer **2B**

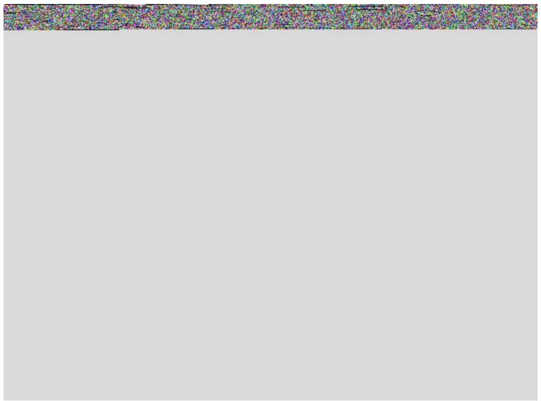

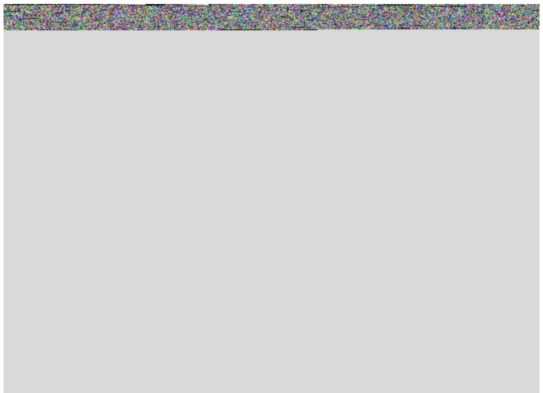

LUMO **HOMO**

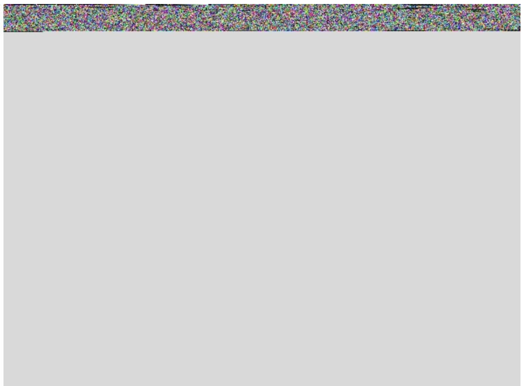

HOMO-1

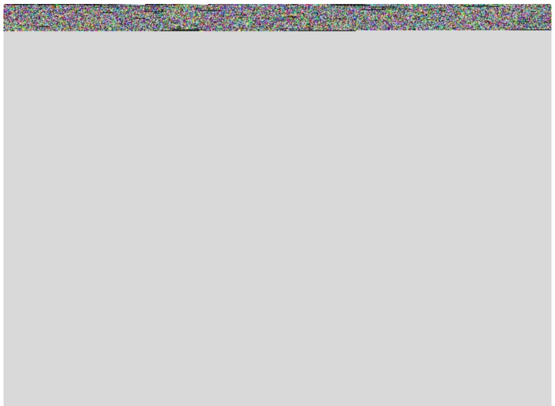

HOMO-2

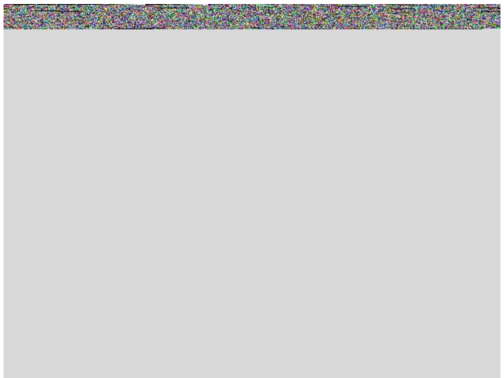

HOMO-3

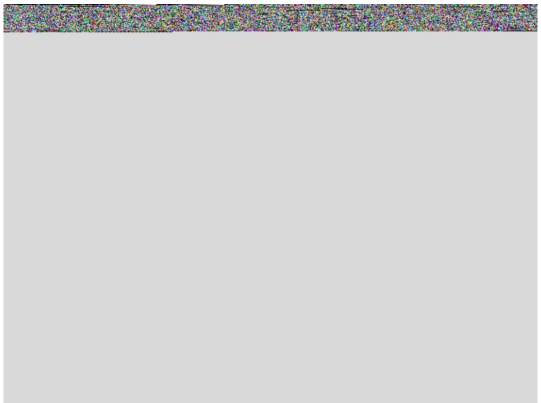

HOMO-4

Figure S23: Frontier Molecular orbital for conformer **2A**
